# Vegetative traits can predict flowering quality in Phalaenopsis orchids despite large genotypic variation in response to light and temperature

**DOI:** 10.1371/journal.pone.0251405

**Published:** 2021-05-11

**Authors:** Evelien van Tongerlo, Wim van Ieperen, Janneke A. Dieleman, Leo F. M. Marcelis

**Affiliations:** 1 Horticulture and Product Physiology, Department of Plant Sciences, Wageningen University & Research, Wageningen, The Netherlands; 2 Greenhouse Horticulture, Wageningen University & Research, Wageningen, The Netherlands; Swedish University of Agricultural Sciences, SWEDEN

## Abstract

*Phalaenopsis* is an economically important horticultural ornamental, but its growth is slow and costly. The vegetative cultivation phase is long and required to ensure sufficient plant size. This is needed to develop high quality flowering plants. We studied the effects of temperature (27 or 31 °C) and light intensity (60 or 140 μmol m^-2^ s^-1^) on plant growth and development during the vegetative cultivation phase in two experiments, with respectively 19 and 14 genotypes. Furthermore, the after-effects of treatments applied during vegetative growth on flowering traits were determined. Increasing light intensity in the vegetative phase accelerated both vegetative plant growth and development. Increasing temperature accelerated vegetative leaf appearance rate, but strongly reduced plant and root biomass accumulation when temperatures were too high. Flowering was greatly affected by treatments applied during vegetative growth, and increased light and temperature increased number of flower spikes, and number of flowers and buds. Genotypic variation was large in *Phalaenopsis*, especially in traits related to flowering, thus care is needed when generalising results based on a limited number of cultivars. Plant biomass and number of leaves during vegetative growth were positively correlated with flowering quality. These traits can be used as an early predictor for flowering capacity and quality of the final product. Additionally, this knowledge can be used to improve selection of new cultivars.

## Introduction

*Phalaenopsis* is an economically important horticultural crop, which is cultivated either as potted ornamental or as cut flower. The potted *Phalaenopsis* accounts for 19 and 32% of the potted plant sales in the USA and Europe, respectively [1]. Growth of *Phalaenopsis* is slow and therefore costly. The natural habitat of *Phalaenopsis* is evergreen forests in tropical and subtropical Asia, characterized by relatively constant humid, warm and relatively shaded conditions. While seasonality hardly occurs in these forests, variations in environmental conditions are sufficient to induce flowering [2]. In commercial cultivation, temperature is the main determinant for different *Phalaenopsis* cultivation phases [3, 4]. Plant development is defined by the rate at which organs (e.g. leaves and flowers) are initiated and appear [5]. An increase in temperature, up to a certain optimum, increases plant development rates and thus affects duration of each cultivation phase.

*Phalaenopsis* cultivation can be divided in three separate phases. The vegetative phase of *Phalaenopsis* is the longest phase, which takes 50–70 weeks on average, measured from the moment that plants move to the greenhouse after propagation in the lab [4, 6]. During this phase, *Phalaenopsis* is grown at high temperatures (≥28°C) which promotes leaf initiation and outgrowth, and inhibit flowering [7]. Flowering in *Phalaenopsis* is mainly temperature-controlled, and temperatures below 25°C induce flowering [8]. In practice, plants in the flower induction phase are exposed to temperatures between 19–21°C for 6–9 weeks [7, 9]. This phase is followed by the flowering phase which lasts approximately 8–10 weeks, in which plants are exposed to higher temperatures (approximately 22°C) to accelerate flower development [8, 9]. Flower induction and flower outgrowth can be relatively well controlled, and most of the research so far has focussed on induction and the process of flowering itself, ranging from environmental factors [10–13] and hormonal control [14, 15] to understanding of the genetic pathways involved in flower development [16, 17]. Although *Phalaenopsis* plants are grown for their flowers, the vegetative cultivation phase is important to ensure sufficient plant size. It is commonly assumed that this is necessary to develop multiple flower spikes and high quality flowers, which increases the plant’s economic value [8]. Per *Phalaenopsis* leaf, two undifferentiated, dormant axillary buds are present. Under favourable environmental conditions, the upper bud can develop into a flower spike once the plant has matured [18]. Therefore, number of leaves is considered an important indicator during vegetative cultivation [6, 12], as flowering potential is thought to increase with number of leaves. Previous studies on *Phalaenopsis* showed that increasing temperature from 28°C to 31°C in the vegetative phase the number of leaves increased, although this did not always result in increased leaf area [19]. Reducing nocturnal temperature resulted in less leaves, lower leaf area and reduced biomass accumulation [20].

Saturating light levels are in the range of 130–200 μmol m^-2^ s^-1^, depending on plant stage, temperature and cultivar [2, 21, 22 and references therein]. Because of these low intensities, light is considered of secondary importance when compared to temperature [11]. However, several studies on *Phalaenopsis* showed that increased irradiance during the vegetative phase resulted in increased leaf initiation rate and leaf area [10, 11, 19]. These traits are also affected by photoperiod and daily light integral (DLI), which promoted leaf growth, leaf initiation rate and biomass accumulation [7, 11]. While a relatively low temperature is considered the main factor for flower induction, a sufficient level of irradiance is also needed for flowering. When light intensities are too low, time to flowering is delayed [23], or even completely absent [13]. Vice versa, higher light intensities in the flowering phase resulted in a reduced time to visible flower spike (flower induction), a higher number of flower spikes, and a higher number of flowers [10, 11, 19]. Time to visible flower spike was positively correlated with higher levels of soluble sugars [23, 24], which suggests that there might be a role for carbohydrates in number of days to spiking.

Thus, both light and temperature are important for various key processes in *Phalaenopsis*, and the two factors interact in ways that are yet poorly understood. To provide insight in how underlying traits contribute to growth and development in *Phalaenopsis*, and how they correlate, a hierarchical component analysis can be used [25]. It helps to systematically study how these components are affected by changes in environmental factors and their interaction. This method has been applied on several other crop species, such as tomato [25], anthurium [26], and wheat and rice [27], and can be used to find desirable characteristics that contribute to either a reduction of vegetative growth time and/or increased plant quality.

The number of studies on young *Phalaenopsis* plants is limited, but considering the lengthiness of the vegetative phase, an improvement might rapidly increase the economy of the cultivation cycle. The need to expand knowledge on young plants is also recognized by e.g. Runkle [7], who calls for more well-described studies with detailed information on temperature, light intensity and spectrum. Optimizing climate conditions in the vegetative phase might result in a reduction of cultivation time and/or in higher quality plants. In addition, there are indications that genotypic variability is significant in these responses. For instance, Dueck *et al*. [19] and Hückstädt and Torre [22] observed genotypic variation in the response of leaf initiation rate, leaf area increase and dry matter accumulation to light intensity. Genotypic variability is also found for the after-effects of treatments in the vegetative phase on flowering [22], and while recognized by others [6, 7] this variation to date remains largely unexplored. Genotypic variability and specific needs are also ignored in practice, where different genotypes are grown in one greenhouse under identical climate conditions.

This study aims to determine the effects of light and temperature on growth and development of *Phalaenopsis* in the vegetative phase, and how treatments in the vegetative phase affect flower induction and flower outgrowth. Furthermore, this study aimed to find whether vegetative traits can be used to predict plant and flower quality in the flowering phase. To increase insight in variability of sensitivity to changes in the environment, this study was conducted on a large set of genotypes. We quantified the contribution of underlying components of plant growth, to determine how these traits correlate with each other. We hypothesized that the vegetative growth stage environment affects reproductive-stage traits and that genotypes vary in their responses to the environment conditions provided, as well as in how sensitive they are to differences in the environment. To determine genotypic variation in the vegetative growth response to light, temperature and their interaction, we conducted two experiments with a broad range of genotypes (19 and 14). The latter experiment (with 14 genotypes) was combined with a follow-up experiment to study the after-effects of light and temperature in the vegetative phase on flower spike growth and quality. Gaining insight in genotypic variability and underlying traits can help to optimize vegetative growth and possible shorten the vegetative cultivation phase. Additionally, this knowledge can be used to improve selection of new cultivars.

## Material and methods

### Plant material

**Fig 1:**
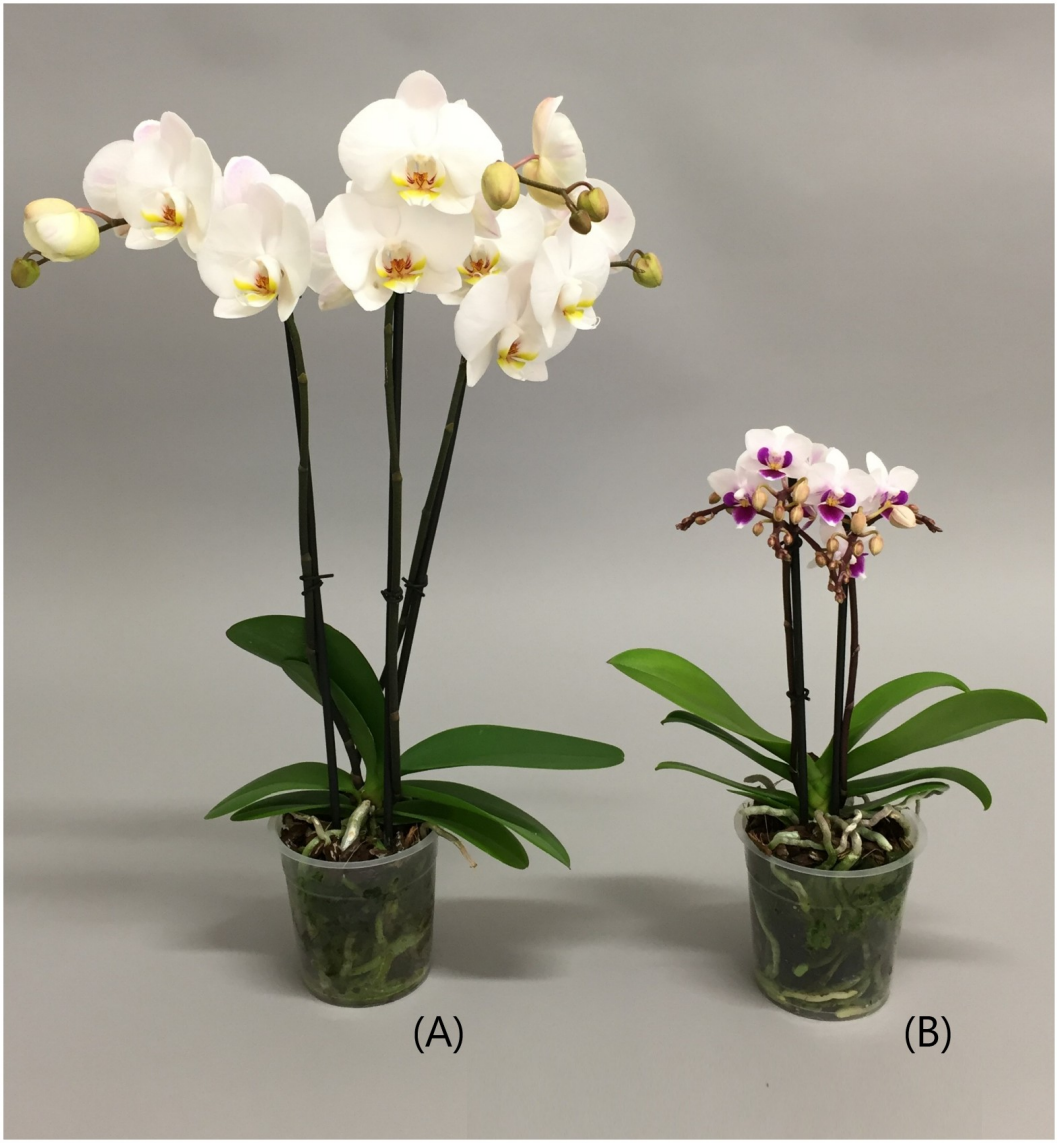
Representative phenotype of *Phalaenopsis* plant types Grandiflora (A) and Multiflora (B).

Vegetative *Phalaenopsis* plants were grown in a Venlo type greenhouse (Bleiswijk, The Netherlands) for 20 weeks after propagation in the lab, before they were transferred to 12 cm transparent pots filled with coconut bark. Genotypes used in this study were provided by breeding company Anthura (Bleiswijk, The Netherlands). Breeding in *Phalaenopsis* focusses on creating either regular sized plants with fewer, but larger flowers and buds (Fig 1A), or smaller plants with a high number of small flowers (Fig 1B) (sometimes referred to as Grandiflora and Multiflora plant types, respectively [28]). In experiment I, 13 Grandiflora and 6 Multiflora genotypes were used, in experiment II, 11 Grandiflora and 3 Multiflora genotypes were used, and the selected genotypes consisted of plants with a variety of flower colours, growth rates, and tendencies to carry one or multiple flower spikes. For detailed information on genotypic similarity and description of phenotypes, see S1 and S2 Files. Genotypes used in experiment II are a subset of experiment I, plus one additional genotype.

### Experimental setup

#### Vegetative phase (Experiment I and II)

Two separate experiments were conducted, but their experimental setup was largely similar (Table 1). Per experiment, two climate chambers were used, corresponding with temperature treatments. In experiment I, light treatments were nested within temperature and replicated three times per climate chamber by creating six separate compartments within the chamber. In experiment II, light treatments were also nested within temperature and replicated twice, by creating four compartments per climate chamber. The second experiment initially aimed to serve as a repetition of temperature, but this experiment was analysed separately because climate conditions were not identical. Experiment II furthermore aimed to increase insight in after-effects of treatments that were applied during vegetative growth on flowering plants.

**Table 1:** Overview of experimental set-up and growth conditions, highlighting differences between setup of vegetative experiment I and II.

	Experiment I	Experiment II
***Experimental setup***		
**Number of genotypes**	19	14
**Transfer to climate chamber**	May	February
**Duration experiment (weeks)**	19	15
***Settings in climate chamber***		
**Temperature (day and night) (°C)**	27	26
	31	30
**Plant density (plants m**^**-2**^**)**	80 (9 weeks)	80
	55 (10 weeks)	
**CO**_**2**_ **concentration (ppm)**	500	800
**Watering interval (days)**	5	7

Experiment I was conducted at Wageningen University & Research in Wageningen, The Netherlands with vegetative plants that were 9 weeks old after transfer to 12 cm pots. Experiment II was conducted at the *Phalaenopsis* breeding company Anthura in Bleiswijk, The Netherlands with vegetative plants that were grown for 5 weeks after transfer to 12 cm pots. Plants were illuminated for 14 hours per day by red/white, and far-red LED modules (Philips LED production module deep red/white and GreenPower LED research module Far Red; Signify, Eindhoven, The Netherlands) at a PPFD of either 60 or 140 μmol m^-2^ s^-1^ and additional far red of 10 or 23 μmol m^-2^ s^-1^, respectively. Here, red light is defined as light between 600 and 700 nm, and far-red as light between 700–800 nm, which resulted in a R:FR ratio of approximately 1.2, or a phytochrome photostationary state of 0.83 [29]. Vapor pressure deficit of the air was set at 1 kPa for all treatments. Plants were watered with nutrient solution with an EC 1.2 mS cm^-1^ and pH of 5.7 (S1 Table).

For destructive harvest in experiment I, 5–7 plants were randomly selected per genotype, per compartment. This resulted in 30–42 plants pooled per temperature treatment. Within temperature, this resulted in 15–21 plants per light treatment. In experiment II, replications within one compartment were treated as being independent, resulting in 20 harvested plants per temperature per genotype, and nested within that 10 plants per light treatment (For a graphic representation of the experimental setup, see S1 Dataset). Vegetative plants were destructively harvested after 19 weeks (experiment I) or 15 weeks (experiment II). In both experiments an initial destructive harvest was conducted (n = 15). Number of leaves and roots, leaf area (LI3100, LiCor, Lincoln, USA), and dry weight of shoot and root were determined. Roots were cut off as close to the stem as possible, and any substrate material that was still attached to the roots was removed. The leaves were then carefully peeled off so the stem remained intact. In the initial harvest, the stem was combined with leaves for dry weight measurements. For all dry weight measurements, plant material was dried for at least 48 hours at 80°C. When plants were transferred to climate chambers, the youngest fully grown leaf of each plant was marked with a clothespin. All leaves that appeared after the leaf with the clothespin were considered new leaves and counted as such. In the final destructive harvest, dry weights of leaves, stem and roots as well as leaf area and number of leaves were determined. Relative growth rate (RGR, g d^-1^) was calculated according to Eq 1, where W represents weight of the plant and t time of harvest. T1 is the initial harvest before start of the experiment. For vegetative plants, this was based on total plant dry weight, but for flowering plants RGR was based on shoot dry weight only. Leaf mass area (lma, g cm^-2^) is the ratio of leaf mass to leaf area, and was calculated using all leaves per plant.

RGR=(ln(W2)-ln(W1))/(t2-t1) (1)

#### Generative phase (continuation of experiment II)

Remaining plants from experiment II continued to grow vegetatively at a lower plant density (60 plants m^-2^) for another eight weeks, before they were moved to the greenhouse (August). Plants were placed at a continuous 19°C (day and night), at a set CO_2_ concentration of 500 ppm, VPD of 0.81 kPa at an average DLI of 7.5 mol m^-2^ day^-1^. To achieve sufficient DLI within a 15 hour day, high pressure sodium (HPS) lighting was switched on towards the end of the day. Plant density was 50 plants per m^-2^, and watering was done every 5–6 days. After eight weeks of flower induction, temperature was set to continuous 20°C, CO_2_ concentration to 650 ppm, while DLI, VPD and watering schedule remained similar. Supplemental lighting was applied both at the beginning and the end of the day. Plants of a genotype were harvested when approximately 2/3 of the plants of that genotype had two open flowers per plant (n = 10). This is called the consumer-ready stage. At this developmental stage plants would normally be sold, and is therefore a good moment to determine plant quality. This means plants were harvested in a physiologically similar developmental stage, rather than an identical point in time. The first genotype was harvested 15 weeks after transfer to the greenhouse, while the last genotype was harvested after 21 weeks. The number of flower spikes, open flowers, buds, total number of leaves, as well as number of new leaves since the start of the experiment, were counted. Leaf area was determined, as well as dry weight of leaves, using the same method as previously described. To calculate RGR of flowering plants, the number of days until harvest per genotype was used.

### Statistical analysis

Data were analysed using linear mixed-effect models using R version 3.6.1 [30] with package lme4 [31]. Measurements of individual plants were pooled per compartment. Treatments and genotype were analysed as fixed effects, and replications of light and temperature were included as random effect as well, because the experimental setup was not full-factorial. The assumption of independent replication of temperature may have underestimated random variance, because technically temperature was not repeated independently per experiment. Therefore, temperature data is analysed for significance at α = 0.01 instead of the commonly used α = 0.05. Residual plot and qqplot were used to determine if assumptions for normality and homogeneity were met. Correlations were tested using Pearson’s correlation coefficient, and visualisation of matrices was done using R-package corrplot v0.85 [32].

## Results

### Vegetative phase (experiment I and II)

**Fig 2:**
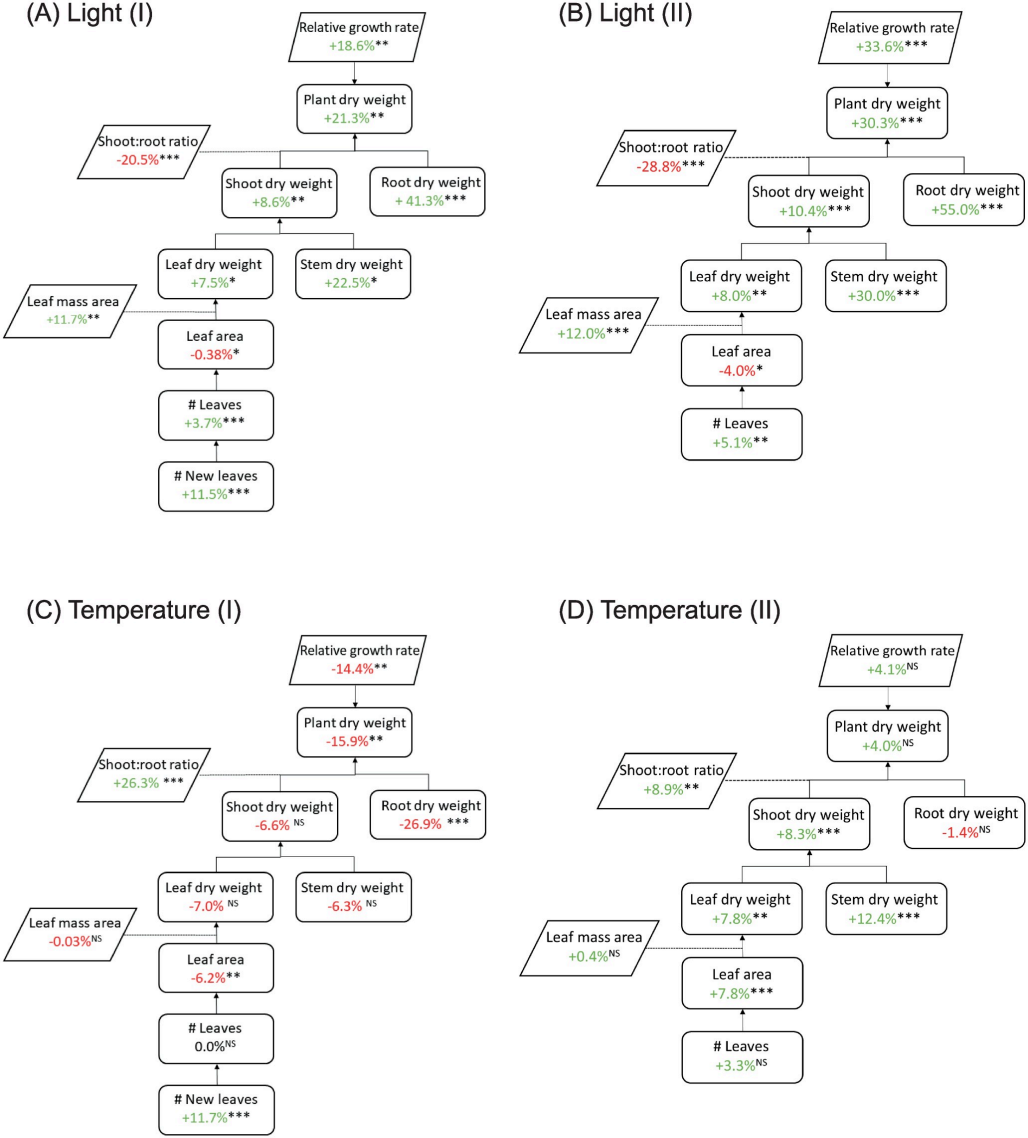
Trait component analysis of vegetative *Phalaenopsis* plants. Main effects of light (A, B) and temperature (C, D). Plants were grown in climate chambers under LED lighting for 14 hours per day at a PPFD of 60 or 140 μmol m^-2^ s^-1^. In experiment I (A,C; n = 3; 5–7 plants per statistical replicate per genotype), plants of 19 genotypes were grown for 19 weeks at either 27°C or 31°C. In experiment II (B,D; n = 5; per genotype) 14 genotypes were grown for 15 weeks at either 26°C or 30°C. Data is averaged over genotypes, percentages represent average change per trait to reference light intensity (60 μmol m^-2^ s^-1^; A, B) or temperature (27°C, C;26° C, D). NS, *,**,*** are not significant or significant at p<0.05, p<0.01 and p<0.001, respectively. For temperature, significance was determined at α<0.01, for light at α<0.05.

The response to light was very similar in both experiments. Total plant biomass increased with an increase in light intensity (Fig 2A and 2B). While both shoot and root dry weight were higher at a higher light intensity, the effect on root dry weight was larger, resulting in a lower shoot:root (S:R) ratio at increased light. In contrast, a higher temperature resulted in higher S:R. Interestingly, in experiment I, root dry weight was the only trait for which the magnitude of the effect of light was temperature-dependent (2-way interaction, p = 0.01), being larger at 27°C than at 31°C (S2 Dataset). In experiment II, this was only the case for stem dry weight. High light intensity resulted in lower leaf area. Number of newly formed leaves, which is considered the most important characteristic needed to shorten growth time, was higher at high light. Leaf mass area (g cm^-2^) was higher under high light, but was not affected by temperature. It appeared that leaves of plants grown at a combination of high light and high temperature were smaller, but plants had more leaves. The opposite is true for plants grown under the combination of low light and a lower temperature, which had fewer, but larger leaves.

Averaged over all genotypes together, total plant dry weight and RGR in experiment I were lower at 31°C than at 27°C (Fig 2C). The response in plant biomass was mainly due to the decrease in root dry weight at higher temperature. Shoot dry weight, composed of leaf and stem dry weight, was not significantly affected by temperature. There was no effect of temperature on plant dry weight or RGR during the vegetative phase in experiment II (Fig 2D). However, shoot dry weight (due to both leaf and stem dry weight) increased with temperature, while root dry weight was not affected. This resulted in an increased S:R ratio in both experiments. In experiment I, the leaf area of plants decreased with an increase in temperature, while number of new leaves increased, although the total number of leaves did not. In experiment II, the number of leaves did not change with temperature either. However, in contrast to experiment I, an increase in temperature resulted in a higher leaf area.

A separate analysis of the data that included only those genotypes that were present in both experiments was conducted (S2 Dataset). This was done to make sure that the differences found between the experiments were not caused solely by the difference in genotypes that were used. A comparative analysis with the original dataset per experiment and the analysis of a subset of genotypes did not result in a deviation from the results as presented in Fig 2.

### Generative phase (Experiment II)

**Fig 3:**
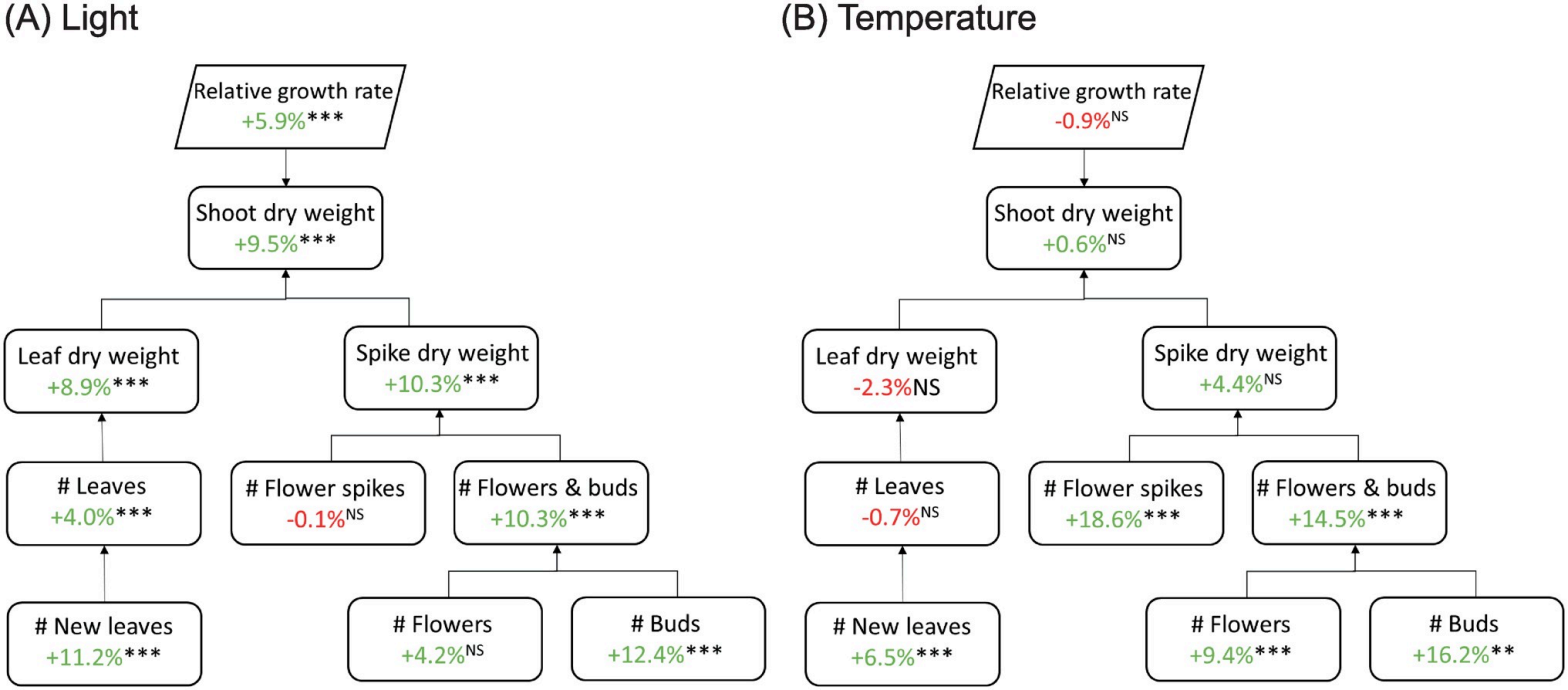
Trait component analysis of flowering *Phalaenopsis* plants. Effects of light (A) and temperature (B) treatments applied during the vegetative phase. During the vegetative phase, plants were grown in climate chambers for 23 weeks at either 26°C or 30°C, and a PPFD of 60 or 140 μmol m^-2^ s^-1^ for 14 hours per day. Plants from all treatments were then moved to the greenhouse for flower induction (8 weeks) and subsequent flowering phase. Plants of a genotype were harvested, when 2/3 reached the consumer-ready stage, defined as plants having two open flowers per plant (15–21 weeks, depending on genotype). Percentages represent average change per trait to either vegetative reference temperature (26°C) or light intensity (60 μmol m^-2^ s^-1^). NS, *,**,*** are not significant or significant at p<0.05, p<0.01 and p<0.001, respectively (n = 10 per genotype). For temperature, significance was determined at α<0.01, for light at α<0.05.

Increasing light intensity from 60 to 140 μmol m^-2^ s^-1^ in the vegetative phase resulted in an increased RGR, which was reflected in both an increased leaf dry weight as well as increased flower spike dry weight (Fig 3A) at the end of the flowering phase. An interaction with temperature occurred for flower spike dry weight (2-way interaction), as increasing temperature increased flower spike dry weight at low light, but not at high light. Additionally, increased light resulted in an increased flower spike quality, defined by the total number of flowers and buds per spike. The increase in number of flowers and buds was due to an increased number of buds. Furthermore, an increased light intensity resulted in a higher leaf number. Similar to spike dry weight, the effect light intensity on number of flower spikes interacted with temperature. An increase in temperature always resulted in a higher number of flower spikes, but this effect was larger for plants grown at low light compared to plants grown at high light during the vegetative phase. There was no main effect of temperature treatments applied during the vegetative stage on biomass-related traits. RGR, shoot and leaf dry weight of the flowering plants were not significantly different (Fig 3B). Flower spike dry weight per plant was slightly higher with increased temperature at the end of the flowering phase, although not statistically different. The number of flower spikes did increase. Increasing temperature during the vegetative stage led to a higher number of new leaves for all genotypes in the flowering stage, as well as to a higher number of flowers and buds per plant.

### Genotypic variability

**Fig 4:**
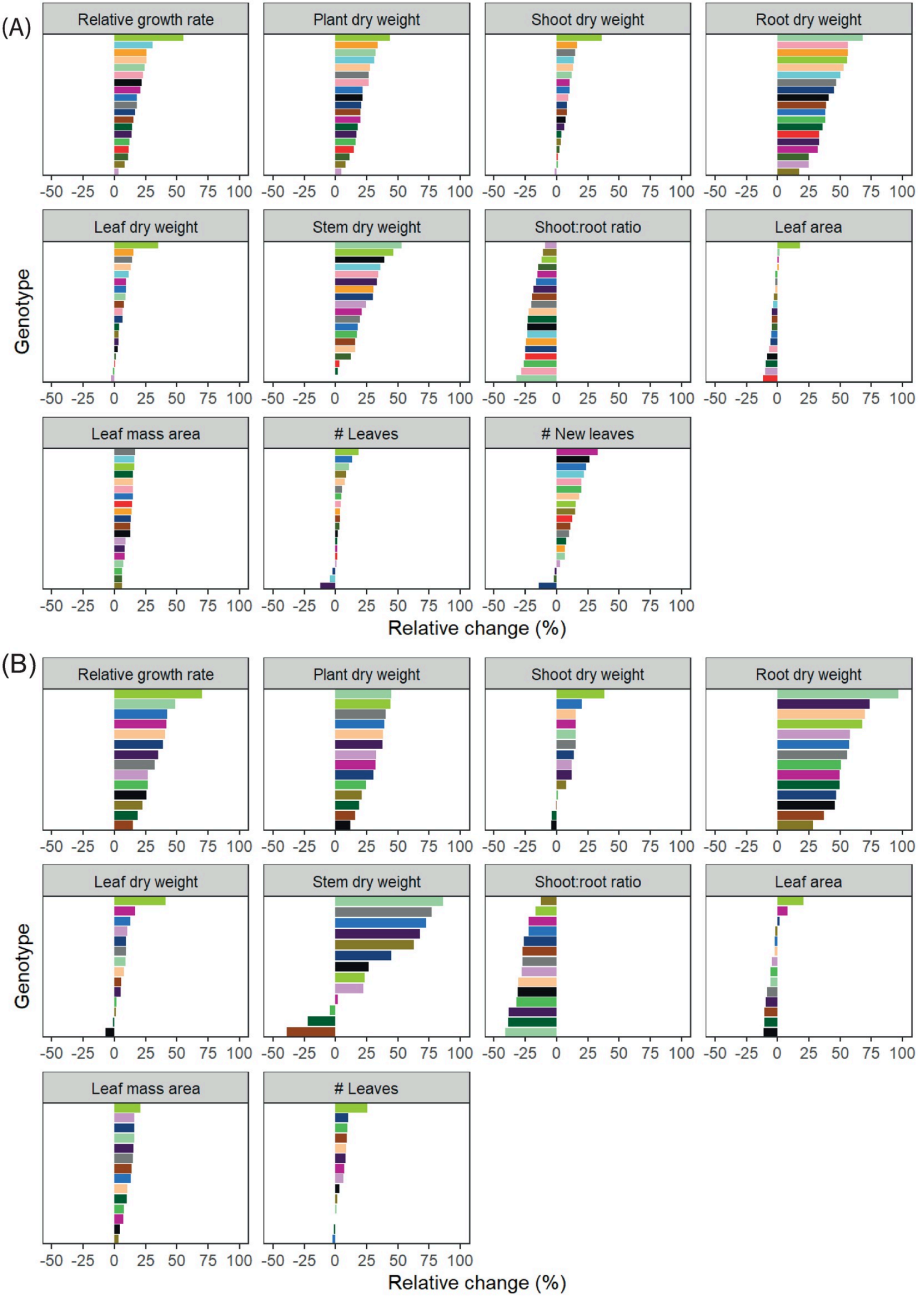
Genotypic variation in vegetative *Phalaenopsis* plants in response to light. Plants were grown in climate chambers under LED lighting for 14 hours per day at a PPFD of 60 or 140 μmol m^-2^ s^-1^. In experiment I (A; n = 3; 5–7 plants per statistical replicate per genotype), plants of 19 genotypes were grown for 19 weeks at either 27°C or 31°C. In experiment II (B; n = 5; per genotype) 14 genotypes were grown for 15 weeks at either 26°C or 30°C. Data is averaged over temperature, and represents relative change per trait to light intensity (60 μmol m^-2^ s^-1^). Similar colours are similar genotypes, also in Figs 5 and 6.

**Fig 5:**
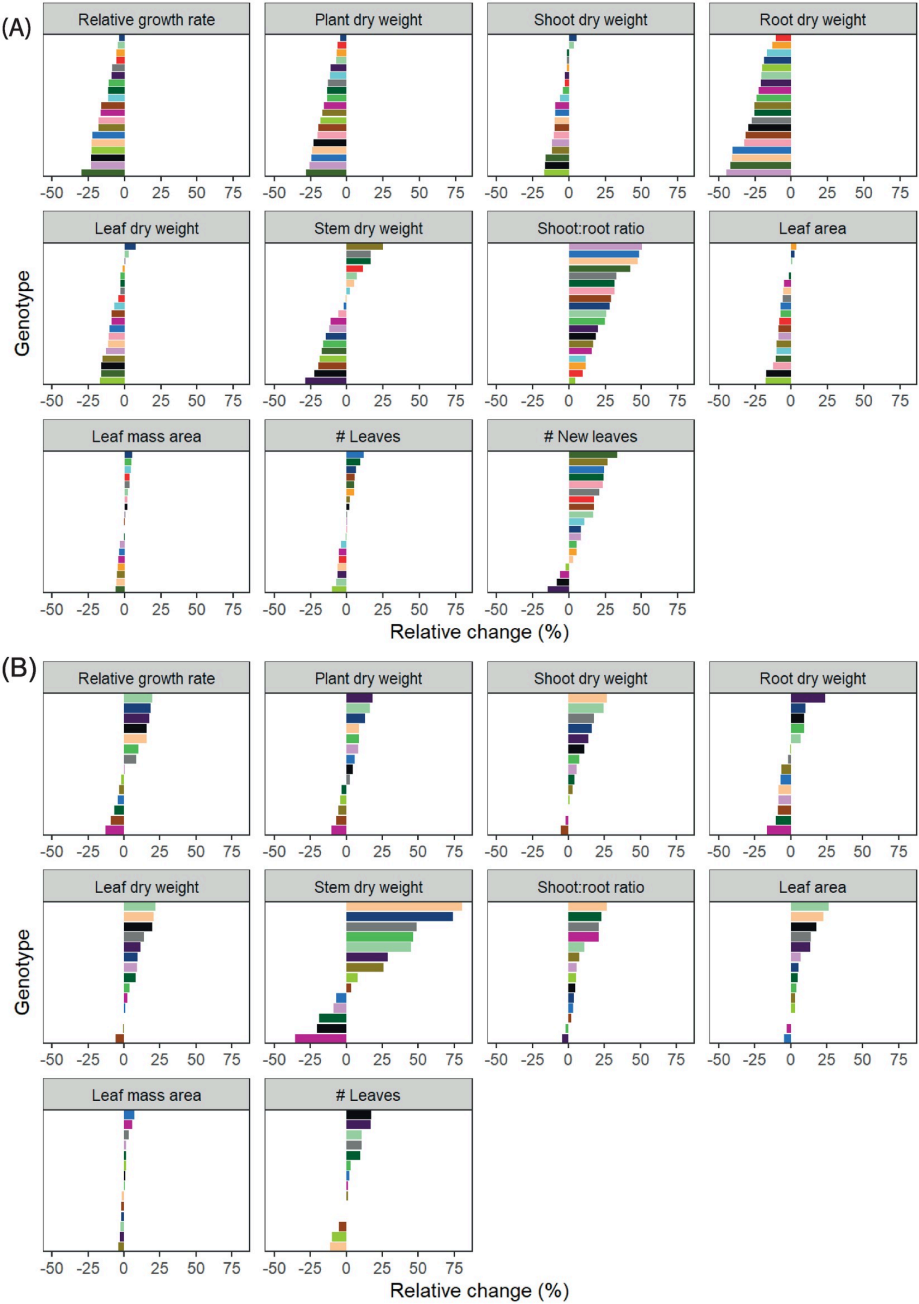
Genotypic variation in vegetative *Phalaenopsis* plants in response to temperature. Plants were grown in climate chambers under LED lighting for 14 hours per day at a PPFD of 60 or 140 μmol m^-2^ s^-1^. In experiment I (A; n = 3; 5–7 plants per statistical replicate per genotype), plants of 19 genotypes were grown for 19 weeks at either 27°C or 31°C. In experiment II (B; n = 5; per genotype) 14 genotypes were grown for 15 weeks at either 26°C or 30°C. Data is averaged over temperature, and represents relative change per trait to light intensity (60 μmol m^-2^ s^-1^). Similar colours are similar genotypes, also in Figs 4 and 6.

The above results are averaged over all genotypes. However, genotypes varied in growth and development, and their individual response to either light or temperature differed, as interaction between genotypes and environmental conditions occurred (Table 2). Significant genotype by environment interactions reflected genotypic variation in the magnitude of responses, and in directionality (i.e. whether genotypes showed an increase or reduction in trait values) (Figs 4 and 5). As described previously, the directionality of the effect of light in both vegetative experiments was comparable, and this was also the case for the range of genotypic variation (Fig 4). While stem dry weight stands out as a trait with large genotypic variation, this is probably due to measurement errors, not to a much higher level of variability and will not be considered in further analyses. In both experiments, least genotypic variation in response to light was found for leaf mass area. Largest variation was found for RGR, which varied over 50% between genotypes.

**Table 2:** Significance of genotypic variation to temperature and light. Effects on vegetative growth traits of 19 (experiment I) or 14 (experiment II) different genotypes of *Phalaenopsis* plants.

	Genotype	Light x genotype	Temperature x genotype
***Experiment I***			
**Relative growth rate**	***^a^	**	***
**Plant dry weight**	***	***	***
**Shoot dry weight**	***	NS	NS
**Root dry weight**	***	***	***
**Leaf dry weight**	***	NS	**
**Stem dry weight**	***	NS	NS
**Shoot:Root ratio**	***	***	***
**Leaf area**	***	**	**
**Leaf mass area**	***	**	***
**Total # leaves**	***	NS	NS
**# new leaves**	***	NS	**
***Experiment II***			
**Relative growth rate**	***	NS	NS
**Plant dry weight**	***	NS	NS
**Shoot dry weight**	***	NS	***
**Root dry weight**	***	NS	NS
**Leaf dry weight**	***	NS	NS
**Stem dry weight**	***	***	***
**Shoot:root ratio**	***	***	NS
**Leaf area**	***	NS	NS
**Leaf mass area**	***	*	NS
**Total # leaves**	***	NS	NS

^a^ Interactions are: NS not significant, or significant at * p<0.05, ** p<0.01 or *** p<0.001. For temperature, significance was determined at α<0.01, for light at α<0.05.

Interestingly, while directionality in response to temperature varies between experiment I and II, the range of genotypic variation was similar (Fig 5), but in experiment II this variation was often not significantly different between genotypes, while this was the case in experiment I (Table 2). In both experiments, least genotypic variation was found for leaf mass area, making it the most stable trait in *Phalaenopsis*. In response to temperature, number of new leaves formed showed largest genotypic variation in experiment I, which varied almost 50% between genotypes. This trait was not measured in experiment II, where largest variation was found in root dry weight, which varied over 40% between genotypes. Overall, genotypic variation was larger in response to light than to temperature.

Also during the flowering phase genotypic variation occurred, as well as interaction with the environment of the vegetative stage (Table 3). More light led to more genotypic variation in the vegetative phase, which can in part be explained by differences between Grandiflora and Multiflora plant types. Genotypic variation for all traits except number of new leaves could be explained by variation between these two plant types. Number of flowers and buds were much more affected by light in Multiflora plants, than was the case for Grandiflora plants (S3 File). The same was true for a change in temperature when the two plant types were compared (Table 3). This was mainly due to a change in number of buds, and not to number of flowers. For the other traits there was no difference between plant types in interaction with the environment. Interaction with the environment mostly came down to variation in the response of individual genotypes, and not to variation between plant types of Grandiflora and Multiflora.

**Table 3:** Effect of plant type and genotypic variation and its interaction with temperature and light in flowering *Phalaenopsis*. Treatments applied in vegetative phase on after-effects during flowering of 14 different genotypes of *Phalaenopsis* plants.

	Plant type	Light x plant type	Temperature x plant type	Genotype	Light x genotype	Temperature x genotype
**Relative growth rate**	***^a^	*	NS	***^a^	***	**
**Shoot dry weight**	***	NS	NS	***	***	**
**Leaf dry weight**	***	NS	NS	***	***	NS
**Flower spike dry weight**	***	NS	NS	***	*	**
**# flower spikes**	***	NS	NS	***	NS	***
**# flowers and buds**	***	*	***	***	**	***
**# buds**	***	***	***	***	***	***
**# flowers**	***	NS	NS	***	***	**
**Total # leaves**	***	NS	NS	***	**	**
**# new leaves**	NS	NS	NS	***	**	NS

^a^ Interactions are: NS not significant, or significant at * p<0.05, ** p<0.01 or *** p<0.001. For temperature, significance was determined at α<0.01, for light at α<0.05.

**Fig 6:**
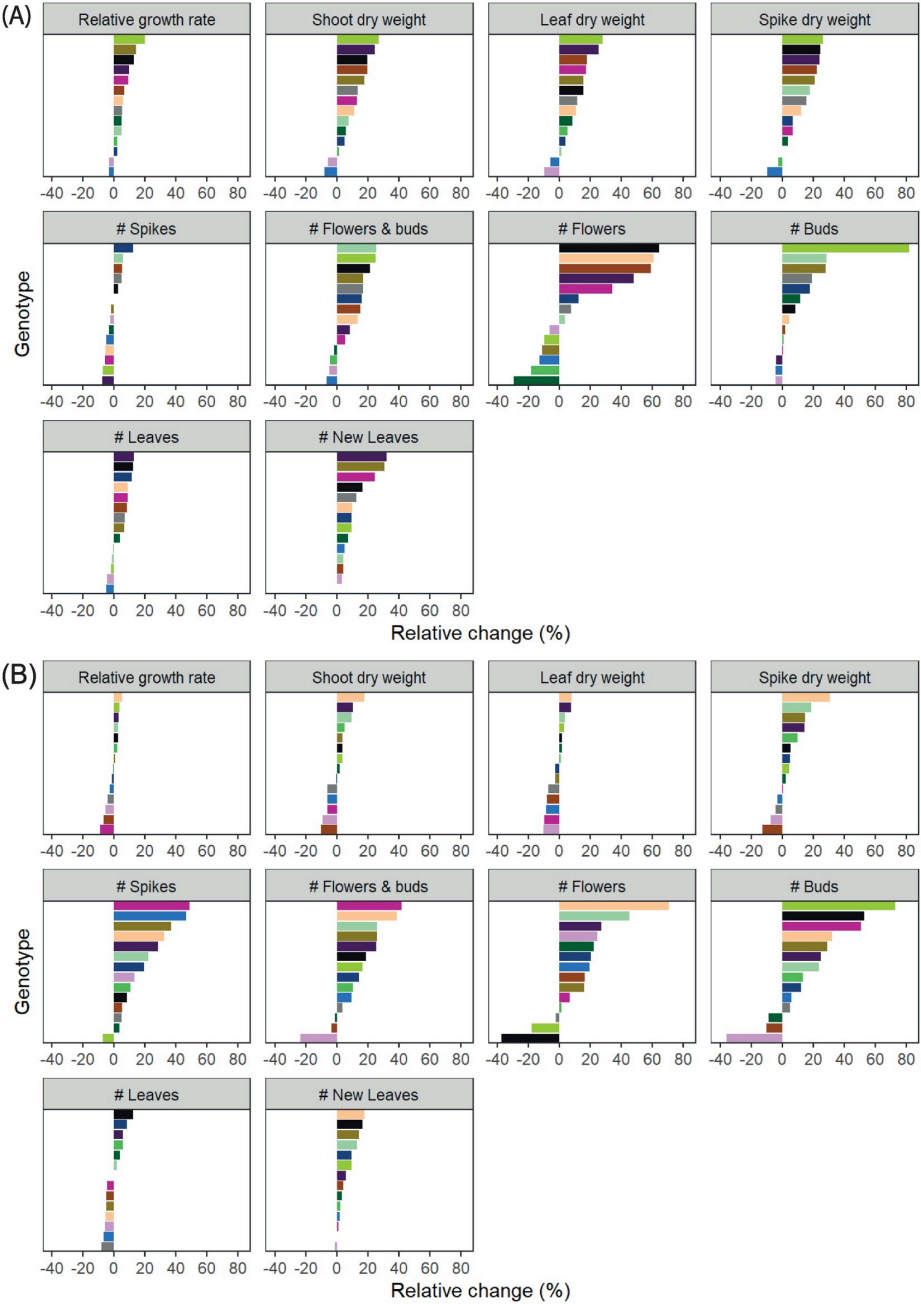
Genotypic variation in flowering *Phalaenopsis* plants of experiment II. During the vegetative phase, plants were grown in climate chambers for 15 weeks at either 26°C or 30°C and a PPFD of 60 or 140 μmol m-2 s-1 for 14 hours per day. Plants from all treatments were simultaneously moved to the greenhouse for cooling and flowering phase until auction-ready; see material and methods for details. Data is averaged either over temperature, and represents relative change per trait to light intensity light intensity (60 μmol m^-2^ s^-1^)(A) or is averaged over light, and represents relative change per trait to temperature (26°C)(B)(n = 10), as applied during vegetative growth. Similar colours are similar genotypes, also in Figs 4 and 5.

Genotypic variation was also apparent in non-flowering traits at the end of the flowering phase, such as leaf dry weight. However, temperature had a larger effect on flower spike dry weight, and even more so on number of flower spikes (Fig 6). There was no significant genotypic variability in number of flower spikes in response to light. Number of flowers and number of buds also showed large genotypic variation, both in response to temperature and to light (Fig 6). This was most likely due to variation in timing of consumer-ready stage between different treatments, as all plants from one genotype were harvested in one batch.

### Correlations between traits

**Fig 7:**
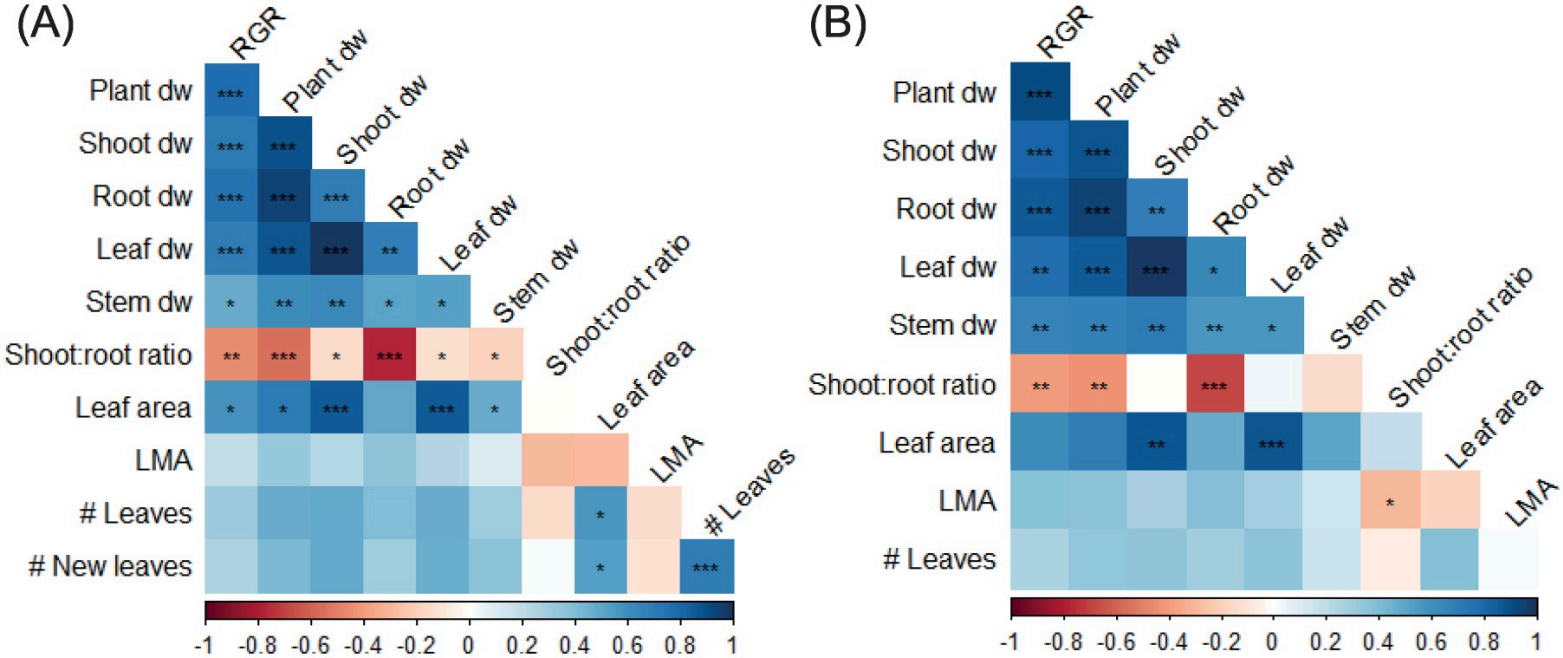
Trait correlation matrix of vegetative *Phalaenopsis* plants. Plants were grown in climate chambers under LED lighting for 14 hours per day at a PPFD of 60 or 140 μmol m^-2^ s^-1^. In experiment I (A; n = 3; 5–7 plants per statistical replicate per genotype), plants of 19 genotypes were grown for 19 weeks at either 27°C or 31°C. In experiment II (B; n = 5; per genotype) 14 genotypes were grown for 15 weeks at either 26°C or 30°C. Data is pooled over genotypes and growth treatment. Colours represent either negative (red) or positive (blue). Significant correlations are marked *,**,*** at p<0.05, p<0.01 and p<0.001, respectively. RGR = relative growth rate, LMA = leaf mass area, dw = dry weight.

Correlations between traits in both vegetative experiments were very consistent (Fig 7). Roots made up the largest part of plant biomass, and therefore had the strongest correlation with total plant dry weight, as well as with shoot:root ratio. RGR was negatively correlated with shoot:root ratio, meaning that shoot:root ratio decreased as RGR increased. Root dry weight changed more with treatments, resulting in a relatively larger impact on these traits. Leaf area and leaf dry weight correlated relatively well for both experiments, although they could not be linked to the number of leaves in experiment II. This suggests that size of individual leaves reduced as number of leaves increased. Over all treatments there was no correlation of any trait with leaf mass area, which was already apparent from previous data (Fig 2).

**Fig 8:**
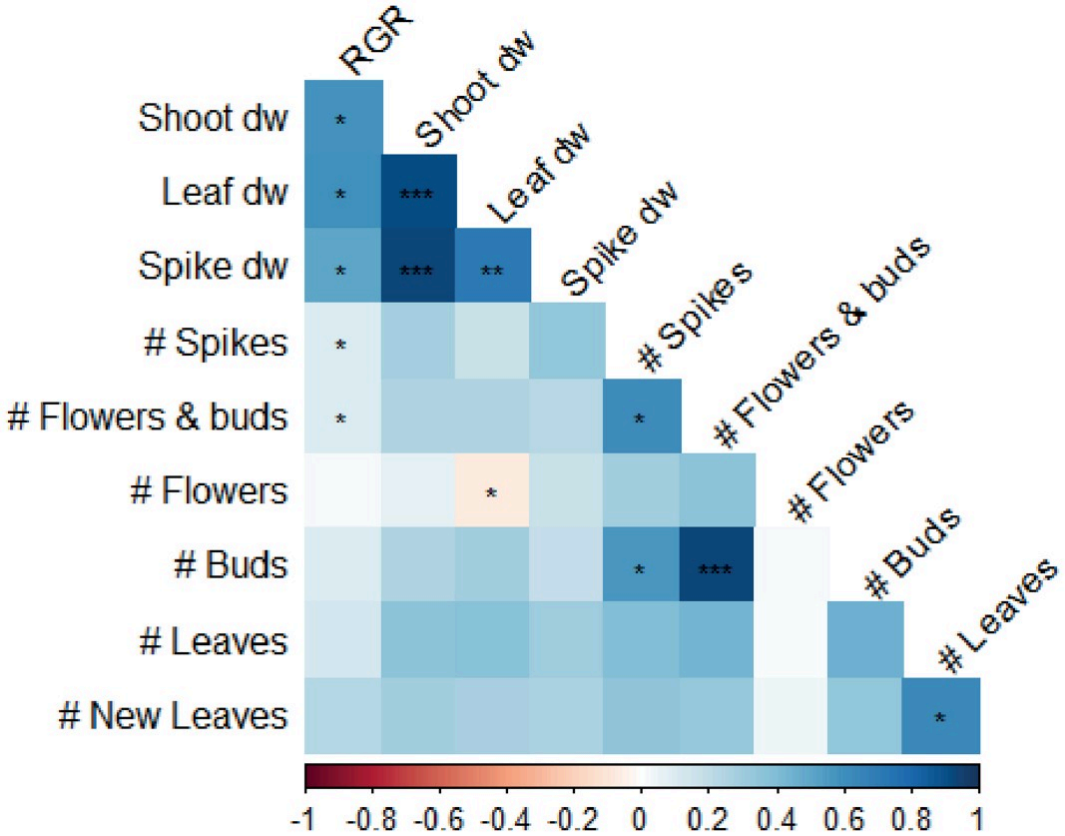
Trait correlation matrix of flowering *Phalaenopsis* plants in experiment II. During the vegetative phase, plants were grown in climate chambers for 23 weeks at either 26°C or 30°C and a PPFD of 60 or 140 μmol m^-2^ s^-1^ for 14 hours per day. Then, plants from all treatments were simultaneously moved to the greenhouse for flower induction and flowering phase until consumer-ready. Data is pooled over genotypes and growth treatment. Colours represent either negative (red) or positive (blue) correlations. Significant correlations are marked *,**,*** at p<0.05, p<0.01 and p<0.001, respectively. RGR = relative growth rate.

**Fig 9:**
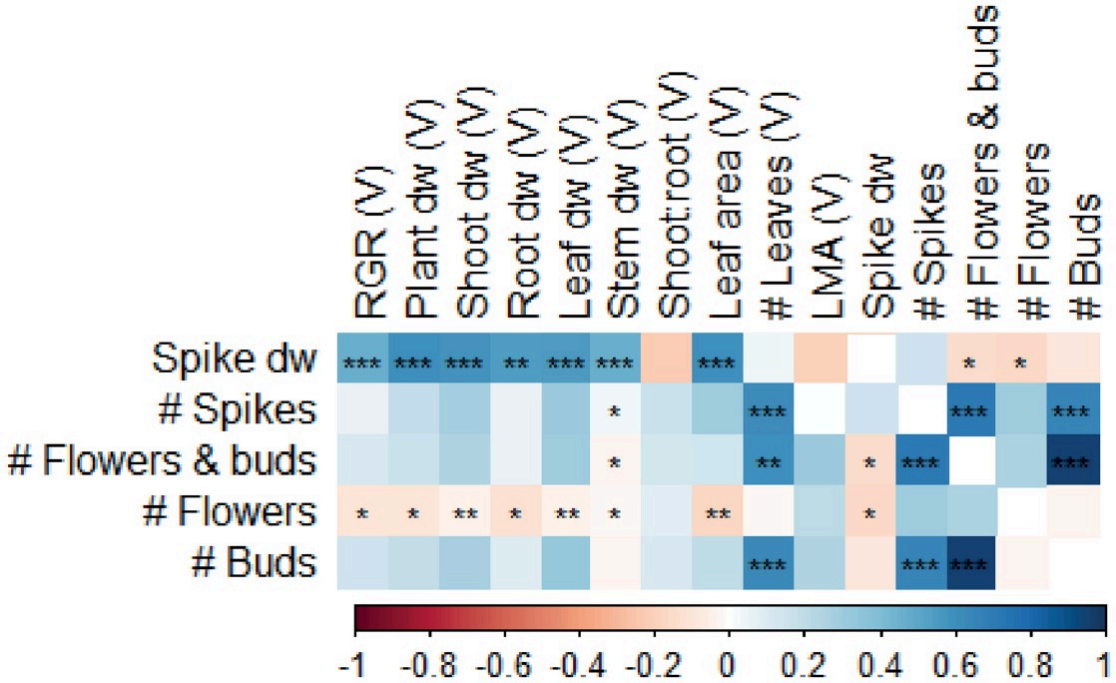
Correlation matrix of vegetative (V) traits with flowering *Phalaenopsis* plants in experiment II. Data is averaged over genotypes and growth treatment. During the vegetative phase, plants were grown in climate chambers for 15 weeks at either 26°C or 30°C and a PPFD of 60 or 140 μmol m^-2^ s^-1^ for 14 hours per day, after which randomly selected plants were harvested. Plants were then allowed to grow for another 8 weeks, before plants from all treatments were simultaneously moved to the greenhouse for flower induction and flowering phase until consumer-ready. Colours represent either negative (red) or positive (blue) correlations. Significant correlations are marked *,**,*** at p<0.05, p<0.01 and p<0.001, respectively. RGR = relative growth rate, LMA = leaf mass area, dw = dry weight.

While an increase in leaf dry weight correlated with an increase in flower spike dry weight, this could not be linked to an increased flower spike number nor to an increase in flowers and buds, indicating that it was the flower spike stem weight itself that increased (Fig 8). Because there were more buds than open flowers (due to time of harvest, at consumer-ready stage), the correlation between total flower potential (number of flowers and buds) was better explained by the number of buds than by the number of flowers. It was expected that more flower spikes resulted in more flowers and buds, but a higher number of flowers and buds could not be correlated to flower spike dry weight. Interestingly, neither the number of leaves nor the number of new leaves of flowering plants was correlated to any of the flowering traits (Fig 8). However, number of leaves in the vegetative phase seemed to correlate well with number of spikes, and number of flowers and buds (Fig 9). Also, a strong correlation of spike dry weight with RGR, plant, shoot, root, leaf and stem dry weight was found.

## Discussion

In this study we have investigated the effects of temperature and light on *Phalaenopsis* plant growth in the vegetative phase, and the after-effects of treatments applied in the vegetative phase on flowering of the plants. An increase in light intensity resulted in an increase in both plant growth and development, visible as increased biomass and plant organ development, i.e. increased number of leaves and roots. The result due to an increase in temperature seemed to depend on the temperature range that was used. The extent to which plant traits were affected by these treatments was genotype-dependent and shows the importance of genotypic variation. Flowers and buds, and number of leaves increased when light intensity and temperature increased. An increased temperature during vegetative growth also resulted in a higher number of flower spikes during flowering. Furthermore, we found that number of leaves in particular correlated well with important flowering traits. The implications of these results are discussed below.

### Increasing light intensity stimulates growth and accelerates development of vegetative *Phalaenopsis* plants

Vegetative growth in *Phalaenopsis* is important, and sufficient vegetative plant size is needed to develop high quality flowering plants [7]. Sufficient plant size is determined by the number of leaves and the plant biomass. With an increase in light intensity in the vegetative phase RGR increased, resulting in more biomass accumulation over time for both shoot and roots (Figs 2 and 3). In particular root biomass was affected strongly by light (Table 2), as additional dry matter was mainly allocated towards roots. Leaves were thicker (more biomass per unit leaf area) under higher light, visible as increased leaf mass area. With an increase in light more leaves were initiated, which is in line with previous results [10, 11]. In this study, increasing light intensity accelerated both growth and development.

### Temperature can increase vegetative plant development but reduces plant growth in supra-optimal range

The effect of light was very similar in both vegetative experiments, whereas the effect of temperature was not. Analysing the data using identical genotypes showed that differences due to temperature treatments between the two experiments were similar to the original analysis with a full range of genotypes per experiment (S2 Dataset). This showed that differences between experiments may be due to the small difference in the range of temperature studied, and are not caused by differences in genotypes used. RGR at high temperature was significantly lower compared to low temperature treatment in experiment I. A lower RGR highly impacted root biomass accumulation (Fig 2). This was not the case in experiment II, where RGR and plant dry weight were not affected by temperature. In both experiments dry matter partitioning changed with temperature, visible as decreased shoot:root ratio (Table 2). A previous study on *Phalaenopsis* found an increase in shoot:root ratio with increasing temperature, although slightly lower night-time temperatures were used [33]. It is difficult to generalize statements on the effect of temperature on shoot:root ratio, as this is very species-dependent and even varies between those sharing the same habitat [34]. *Phalaenopsis* employs crassulacean acid metabolism (CAM), a specialized photosynthetic pathway that temporally separates CO_2_ uptake from CO_2_ decarboxylation. Friemert *et al*. [35] showed that temperature directly affects efflux of malic acid and decarboxylation of CO_2_ in CAM plants due to changes in membrane stability, which might result in CO_2_ leaking out of the leaf. Furthermore, processes such as respiration, enzyme activity and stomatal movement are affected by changes in temperature, even though they might be subject to acclimation [36]. Jeong *et al*. [37] found that in *Phalaenopsis*, high temperature treatments, decreased relative chlorophyll content and CO_2_ uptake. This might explain the lower RGR and thus lower biomass accumulation over time in our current study. It is interesting that RGR and subsequently, plant biomass accumulation are reduced at higher temperatures, considering the native habitat of these plants, where daytime temperatures of 31°C are not exceptional [38]. Blanchard and Runkle [39] showed that with a sufficiently high day temperature (>26°C), plants remain vegetative regardless of temperature during the night, and while lower night temperatures have a positive effect on CO_2_ uptake [40], it is unclear how this affects plant growth and development exactly. Total number of leaves did not change with temperature, but number of newly formed leaves increased in experiment I. However, leaf area decreased with an increase in temperature in experiment I (Fig 2). This might be due to early abscission of old leaves in experiment I. Sufficiently high temperatures accelerates plant growth and development in the vegetative phase [7], but finding the optimal temperature may not be straight forward. The optimal temperature range in *Phalaenopsis* appears to be quite narrow: too low induces premature flowering in the vegetative phase, and although higher temperatures increases development of new leaves, it also leads to reduced growth and appears to accelerate aging and senescence of older leaves.

### Light and temperature treatments in the vegetative phase affect flowering plant growth and development

After-effects of increased light intensity in the vegetative phase were clearly visible in flowering plants. These plants had more leaf and flower spike biomass, and an increased number of leaves, flowers and buds, again indicating that light affects not only growth, but also plant development. Despite an increased number of leaves, the total number of flower spikes was not affected by light in the vegetative phase (Fig 3). Lee *et al*. [11] found an increased number of flower spikes with increased light intensity. However, these increased light intensities were applied during flower induction and flower outgrowth phases as well, not only during the vegetative phase. A different light spectrum during the cooling and flowering phase can increase number of flower spikes [41, 42]. To what extent light quality during vegetative growth affects flowering later on remains to be seen.

There was no main effect of temperature applied in the vegetative phase on above ground plant biomass in the flowering phase (Table 3), but when looking at differences between genotypes, variation occurred in both leaf and flower spike biomass (Fig 6). Traits related to development of organs (i.e. leaf, flower spike and flower number) increased when higher temperatures were applied in the vegetative phase. Interestingly, the effect of temperature on plant developmental rates in the vegetative phase translates to the flowering phase as well. It seems that the exact temperature given determines what is concluded, because Jeong *et al*. [37] found that an increase in temperature can lead to lower number of flower buds, and even to a reduction in number of flower spikes after high temperature stress in the vegetative phase (34°C). Biomass production and carbohydrates play a role in flower spike outgrowth and development [10, 12]. For instance, increasing ambient CO_2_ during the flower induction and flowering phase in *Phalaenopsis* was found to increase number of flowers and flower spikes [43, 44]. Several studies found that floral development and time to visible flower spike is positively correlated to the amount of soluble sugars in the leaves, sucrose in particular [23, 24, 37]. Sucrose levels can be increased directly via photosynthesis or exogenous sucrose application [24], but also indirectly via light spectrum [8] or via application of plant hormones [14], although none of these can completely substitute a low temperature treatment. From these studies it might appear that sucrose content readily available in the leaves and a continuous supply to the reproductive bud determines flower potential in the end, but sucrose alone is not the signalling factor for flower induction [15, 45]. *Phalaenopsis* does not have storage organs such as pseudobulbs [2], but it might be that long-term storage of assimilates does take place which can be used later on during flowering. It remains unclear exactly how number of flower spikes and number of flowers and buds are affected by treatments applied in the vegetative phase, but there is a strong correlation between vegetative growth and flowering traits (Fig 9), which highlights the importance of studying vegetative growth in *Phalaenopsis*.

### Importance of genotypic variability

Within main effects of temperature and light intensity on growth and flowering in *Phalaenopsis*, genotypic variation was observed. For most traits there was an interaction of light x genotype, and/or temperature x genotype (Table 2, Figs 4 and 5). Traits such as shoot and leaf dry weight were not significantly different on main effect level in experiment I, but they were different when genotype was considered. For instance, RGR was strongly affected by an increase in light in one genotype, but when it comes to an increase in leaves, that same genotype was performing average (Fig 4A). The genotype that had the largest increase in number of leaves with an increase in light intensity in experiment II in the vegetative phase (Fig 4B), was negatively affected by temperature (Fig 5B). Furthermore, in this genotype high temperature even resulted in a reduced number of flower spikes at the end of the flowering phase (Fig 6A). Results like these confirm the importance of including a large number of genotypes when studying Phalaenopsis growth and development [7, 22]. Our study shows that genotypic variation for flowering traits is large (Fig 6) and what is true for one genotype, might not hold for another. The effect of increased light in the vegetative phase on number of flower spikes was not genotype dependent in this study, but Hückstädt and Torre [22] did find genotypic variability in response to light when two genotypes were compared. Working with a larger set of 14 genotypes showed that the number of flower spikes in most genotypes is probably not affected by light intensity in the vegetative phase. Furthermore, an increase in temperature in the vegetative phase increased number of flower spikes up to 50% in some genotypes, while it led to a decrease in others (Fig 6B), which might therefore explain seemingly contradictory results in the literature (e.g. [37]). Detailed information on genotypic variability can also be used as the basis for decisions on growing strategies. For instance, it might be cost-effective to invest in supplemental lighting when genotypes that respond strongly to an increase in light intensity are being cultivated, but not in those that are hardly affected.

### Vegetative traits as predictor for flowering quality

In both experiments and regardless of treatment, an increase in RGR in the vegetative phase correlated with a decrease in shoot:root ratio (Fig 7), emphasizing the impact of root biomass on *Phalaenopsis* plant growth. This makes sense, considering that in epiphytic plants the root system is highly important, as it plays a large role in water and nutrient absorption [46]. However, roots are often overlooked when it comes to traits that are considered relevant in breeding and production of *Phalaenopsis*, but it might be that root-related traits can be correlated with flower induction and flowering potential. It is generally assumed that bigger plants with more leaves result in a higher number of flower spikes, as well as a higher number of flowers per flower spike. This assumption is based on the fact that two bud primordia for flower spikes are differentiated at the base of each leaf [47]. Flower spikes are most likely to appear from the 3th or 4^th^ node [12]. While this might still be true, leaf number alone does not guarantee a certain number of flower spikes, as flower induction is also affected by other factors [7, 8, 15, 45]. Hückstädt and Torre [22] found no relation between number of leaves and number of flower spikes on two *Phalaenopsis* genotypes studied. In our study with 14 genotypes and a combination temperature and light treatments we did find a correlation between traits measured in the vegetative phase, and traits during flowering (Fig 9). Number of leaves was positively correlated with number of flower spikes, and with number of flowers and buds. Thus, increasing leaf initiation rates during the vegetative phase leads to higher flowering plant quality. Here, this was done by changing light and temperature treatments. For *Doritaenopsis*, elevated CO_2_ during vegetative growth increased CO_2_ uptake and leaf initiation [48]. Increased CO_2_ during vegetative and flowering phases in *Phalaenopsis* resulted in more branching and more flowers, indicating that flowering quality increased [44]. Linking data of vegetative plants grown at different environmental conditions with flowering characteristics later on, can assist in the selection of new cultivars during breeding, as it can be used as an early predictor for flowering capacity and quality.

## Conclusion

We studied the interaction between temperature and light on growth and development of *Phalaenopsis*, so that leaf initiation rate and dry matter production would be optimized. These traits in particular were considered important in the vegetative phase, as they would result in higher quality flowering plants. This study has led to several new insights. 1) Increasing light intensity accelerates both plant growth and development in *Phalaenopsis*. 2) Increasing temperature can accelerate plant development, but can quickly lead to reduced growth when supra-optimal. 3) Genotypic variation in the response to temperature and light is large in *Phalaenopsis*, especially in traits related to flowering. Therefore, sufficient genotypic variation in studies is important and care is needed when generalising results. 4) Growth in the vegetative phase can be linked to flowering traits. The positive correlation between number of leaves during the vegetative phase and number of flower spikes can be used to predict flowering capacity and quality of the final product.

## Supporting information

S1 File Phenotypic description of Phalaenopsis genotypes. Genotype 1,2 and 4–20 were used in experiment I, and genotypes 1–14 were used in experiment II.
(DOCX)

S2 File Genotypic variation. Genotypic similarity in this study was determined based on the variety tracer method, developed by NAKtuinbouw (Roelofarendsveen, The Netherlands) to identify plant Phalaenopsis varieties. This is done based on the presence or absence of different alleles for 8 SSR markers (H. Teunissen, personal communication). This information was then used to create a similarity matrix based on the Jaccard coefficient. To further display genotypic variation, PCA was done and a dendrogram created that shows the variation of the genotypes used in this study relative to the complete genotypic pool of the breeder, from which plants were acquired.
(DOCX)

S3 File Genotypic variation in flowering Phalaenopsis based on plant type. (DOCX)

S1 Table Composition of nutrient solution. (DOCX)

S1 Dataset Experimental setup and raw data—Effect of light and temperature in the vegetative phase on Phalaenopsis plants. Plants were grown in climate chambers under LED lighting for 14 hours per day at a PPFD of 60 or 140 μmol m^-2^ s^-1^. In experiment I, plants of 19 genotypes were grown for 19 weeks at either 27°C or 31°C, before harvest. In experiment II, 14 genotypes were grown for 15 weeks at either 26°C or 30°C, after which a random selection of plants were harvested (“vegetative”). Remaining plants were then allowed to grow for another 8 weeks, before plants from all treatments were simultaneously moved to the greenhouse for flower induction and flowering phase until consumer-ready, after which a second harvest was conducted (“flowering”).
(XLSX)

S2 Dataset Statistical analysis. Anova of the linear mixed-models conducted for trait components, genotypic variation, subsets of genotypes present in both experiments, and plant type, as well as Pearson’s correlation coefficient and analysis for correlation matrices.
(XLSX)

## References

[1] Davis SC, Simpson J, del Gil Vega, Niechayev NA, van Tongerlo E, Castano NH, et al. Undervalued potential of crassulacean acid metabolism for current and future agricultural production. J Exp Bot. 2019;70(22):6521–37. 10.1093/jxb/erz223 31087091 PMC6883259

[2] Christenson EA. Phalaenopsis—A monograph. Portland, Oregon: Timber Press; 2001.

[3] Blanchard M, Lopez R, Runkle ES, Wang Y-T. Growing the Best Phalaenopsis. Orchids. 2007;4:266–71. Available from: https://www.aos.org/AOS/media/Content-Images/PDFs/GrowingtheBestPhalaenopsisPart_4.pdf.

[4] van der Knaap N. Cultivation Guide Phalaenopsis: Knowledge for Professionals. Anthura; 2005.

[5] Atkinson D, Porter JR. Temperature, plant development and crop yields. Trends Plant Sci. 1996;1(4):119–24.

[6] Paradiso R, De Pascale S. Effects of plant size, temperature, and light intensity on flowering of Phalaenopsis hybrids in mediterranean greenhouses. Sci World J. 2014. 10.1155/2014/420807 PMC4258309 25506068

[7] Runkle ES. Environmental control of the flowering process of Phalaenopsis orchids. Acta Hortic. 2019;1262:7–12.

[8] Dueck T, Trouwborst G, Hogewoning SW, Meinen E. Can a high red: Far red ratio replace temperature-induced flower spike development in Phalaenopsis? Environ Exp Bot. 2016;121:139–44.

[9] Paradiso R, Maggio A, De Pascale S. Moderate variations of day/night temperatures affect flower induction and flower spike development in Phalaenopsis. Sci Hortic (Amsterdam). 2012;139:102–7.

[10] Konow EA, Wang YT. Irradiance levels affect in vitro and greenhouse growth, flowering, and photosynthetic behavior of a hybrid Phalaenopsis orchid. J Am Soc Hortic Sci. 2001;126(5):531–6.

[11] Lee HB, Lee JH, An SK, Park JH, Kim KS. Growth characteristics and flowering initiation of Phalaenopsis Queen Beer ‘Mantefon’ as affected by the daily light integral. Hortic Environ Biotechnol. 2019;60(5):637–45.

[12] Sakanishi Y, Imanishi H, Ishida G. Effect of Temperature on Growth and Flowering of Phalaenopsis amabilis. Bull Univ Osaka Prefect Ser B, Agric Biol. 1980;32:1–9.

[13] Wang Y-T. Phalaenopsis orchid light requirement during the induction of spiking. HortScience. 1995;30(1):59–61.

[14] Blanchard M, Runkle ES. Benzyladenine promotes flowering in Doritaenopsis and Phalaenopsis orchids. J Plant Growth Regul. 2008;27(2):141.

[15] Chen W, Liu H, Liu Z, Yang L, Chen W. Gibberllin and temperature influence carbohydrate content and flowering in Phalaenopsis. Physiol Plant. 1994;90(2):391–5.

[16] Wang SL, Viswanath KK, Tong CG, An HR, Jang S, Chen FC. Floral Induction and Flower Development of Orchids. Front Plant Sci. 2019;10:1–15. 31649713 10.3389/fpls.2019.01258 PMC6795766

[17] Wang HM, Tong CG, Jang S. Current progress in orchid flowering/flower development research. Plant Signal Behav. 2017;12(5). 10.1080/15592324.2017.1322245 28448202 PMC5501233

[18] Hsiao Y-Y, Pan Z-J, Hsu C-C, Yang Y-P, Hsu Y-C, Chuang Y-C, et al. Research on orchid biology and biotechnology. Plant Cell Physiol. 2011;52(9):1467–86. 10.1093/pcp/pcr100 21791545

[19] Dueck T, de Boer P, van Noort F. Teeltversnelling Phalaenopsis door klimaatoptimalisatie tijdens op- en afkweek [Internet]. 2011. https://edepot.wur.nl/163349.

[20] Pollet B, Kromwijk A, Vanhaecke L, Dambre P, Van Labeke M, Marcelis LFM, et al. A new method to determine the energy saving night temperature for vegetative growth of Phalaenopsis. Ann Appl Biol. 2011;158:331–45.

[21] Lootens P, Heursel J. Irradiance, temperature, and carbon dioxide enrichment affect photosynthesis in Phalaenopsis hybrids. HortScience. 1998;33(7):1183–5.

[22] Hückstädt AB, Torre S. Irradiance during Vegetative Growth Phase Affects Production Time and Reproductive Development of Phalaenopsis. Eur J Hortic Sci. 2013;78(4):160–8.

[23] Kataoka K, Sumitomo K, Fudano T, Kawase K. Changes in sugar content of Phalaenopsis leaves before floral transition. Sci Hortic (Amsterdam). 2004;102(1):121–32.

[24] Lee HB, Jeong SJ, Lim NH, An SK, Kim KS. Correlation between carbohydrate contents in the leaves and flower spike initiation in Phalaenopsis. Sci Hortic (Amsterdam). 2020;265:109270.

[25] Higashide T, Heuvelink E. Physiological and Morphological Changes Over the Past 50 Years in Yield Components in Tomato. J Am Soc Hortic Sci. 2009;134(4):460–5.

[26] Li T, Heuvelink E, Van Noort F, Kromdijk J, Marcelis LFM. Responses of two Anthurium cultivars to high daily integrals of diffuse light Scientia Horticulturae Responses of two Anthurium cultivars to high daily integrals of diffuse light. Sci Hortic (Amsterdam). 2014;179:306–13.

[27] Cai C, Yin X, He S, Jiang W, Si C, Paul C. Responses of wheat and rice to factorial combinations of ambient and elevated CO 2 and temperature in FACE experiments. Glob Chang Biol. 2016;22:856–74. 10.1111/gcb.13065 26279285

[28] Chen C, Lin R-S. Nondestructive estimation of dry weight and leaf area of Phalaenopsis leaves. Appl Eng Agric. 2004;20(4):467–72.

[29] Sager JC, Smith WO, Edwards JL, Cyr KL. Photosynthetic Efficiency and Phytochrome Photoequilibria Determination Using Spectral Data. Trans ASAE. 1988;31:1882–9.

[30] R Core Team. R: A Language and Environment for Statistical Computing [Internet]. Vienna, Austria; 2019. https://www.r-project.org

[31] Bates D, Mächler M, Bolker B, Walker S. Fitting Linear Mixed-Effects Models Using {lme4}. J Stat Softw. 2015;67(1):1–48.

[32] Wei T, Simko V. R package “corrplot”: Visualization of a Correlation Matrix [Internet]. 2018. https://github.com/taiyun/corrplot.

[33] Chen C. Application of growth models to evaluate the microenvironmental conditions using tissue culture plantlets of Phalaenopsis Sogo Yukidian ‘V3’. Sci Hortic (Amsterdam). 2015;191:25–30.

[34] Luo H, Xu H, Chu C, He F, Fang S. High Temperature can Change Root System Architecture and Intensify Root Interactions of Plant Seedlings. Front Plant Sci. 2020;11(160):1–13. 10.3389/fpls.2020.00160 32161613 PMC7054236

[35] Friemert V, Heininger D, Kluge M, Ziegler H. Temperature effects on malic-acid efflux from the vacuole and on the carboxylation pathways in Crassulacean-acid-metabolism plants. Planta. 1988;174:453–61. 10.1007/BF00634473 24221560

[36] Lüttge U. Ecophysiology of Crassulacean Acid Metabolism (CAM). Ann Bot. 2004;93(6):629–52. 10.1093/aob/mch087 15150072 PMC4242292

[37] Jeong SJ, Lee HB, An SK, Kim KS. High temperature stress prior to induction phase delays flowering initiation and flower spike development in Phalaenopsis queen beer ‘Mantefon’. Sci Hortic (Amsterdam). 2020;263:109092.

[38] Pridgeon AM. The Illustrated Encyclopedia of Orchids. Portland, Oregon, USA: Timber Press; 2000.

[39] Blanchard M, Runkle ES. Temperature during the day, but not during the night, controls flowering of Phalaenopsis orchids. J Exp Bot. 2006;57(15):4043–9. 10.1093/jxb/erl176 17075080

[40] Pollet B, Storme E, Steppe K, Lemeur R, Van Labeke MC, Dambre P. Determining the optimal nighttime air temperature for phalaenopsis during the vegetative stage. Acta Hortic. 2011;893:865–72.

[41] Magar YG, Noguchi A, Furufuji S, Kato H, Amaki W. Effects of light quality during supplemental lighting on Phalaenopsis flowering. Acta Hortic. 2019;1262:75–9.

[42] Magar YG, Koshioka S, Noguchi A, Amaki W. Effects of light quality during cultivation on the flowering and floret arrangement in Phalaenopsis amabilis. Acta Hortic. 2020;1271:135–9.

[43] Dueck T, Meinen E, Kromwijk A. Nachtbelichting en CO2-dosering bij Phalaenopsis [Internet]. 2008. https://edepot.wur.nl/5354.

[44] Trouwborst G, Hogewoning SW, Persoon SH, de Jong A, Sanders J, van der Spek R. Zuiniger met CO2 bij gelijkblijvende of hogere productie? 2016.

[45] Qin Q, Kaas Q, Zhang C, Zhou L, Luo X, Zhou M, et al. The Cold Awakening of Doritaenopsis “Tinny Tender” Orchid Flowers: The Role of Leaves in Cold-induced Bud Dormancy Release. J Plant Growth Regul. 2012;31(2):139–55.

[46] Zotz G. Physiological Ecology. In: Plants on Plants—The Biology of Vascular Epiphytes. Cham: Springer International Publishing; 2016. p. 95–148.

[47] Rotor GB. Daylength and temperature in relation to growth and flowering of orchids. Cornell Univ Agric Exp Stn. 1952;885:47.

[48] Yun DL, Kim HJ, Kim YJ. CO2 enrichment increased leaf initiation and photosynthesis in Doritaenopsis Queen Beer ‘Mantefon’ orchids. Hortic Environ Biotechnol. 2018;59(2):159–65.

