# Supplementary material for: Vegetative traits can predict flowering quality in Phalaenopsis orchids despite large genotypic variation in response to light and temperature
Source: PLoS One. 2021 May 11;16(5):e0251405. doi: 10.1371/journal.pone.0251405 (PMC8112652; doi:10.1371/journal.pone.0251405)
Supplement: S1 File — Genotype 1,2 and 4–20 were used in experiment I, and genotypes 1–14 were used in experiment II. (DOCX) [file pone.0251405.s001.docx]

**S1 File. Phenotypic description of *Phalaenopsis* genotypes.** Genotype 1,2 and 4-20 were used in experiment I, and genotypes 1-14 were used in experiment II

| Genotype  number | Color | Picture | Specific characteristics of each genotype | Cultivation time (weeks) | Leaf initiation (leaf week^-1^) | Number of flower spikes | Height of flowering plant (cm) |
| --- | --- | --- | --- | --- | --- | --- | --- |
| 1 | White | 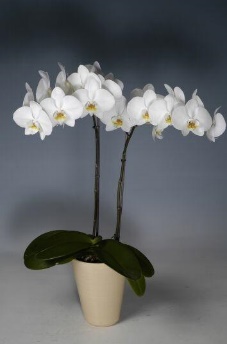 | Reference genotype | 66.37 | 0.110 | 2.06 | 61.83 |
| 2 | White | 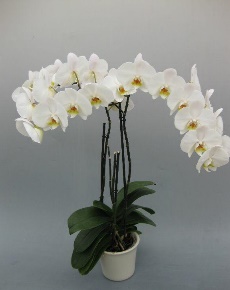 | Highest leaf initiation rate and highest number of flower spikes | 64.14 | 0.144 | 3.08 | 58.57 |
| 3 | White with pink stripe | 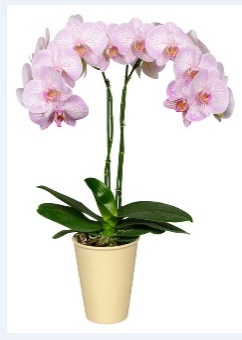 | Low number of flower spikes compared to number of leaves (opposite of genotype 7). Big difference in summer and winter growth | 69.67 | 0.094 | 1.78 | 63.33 |
| 4 | White, red lip | 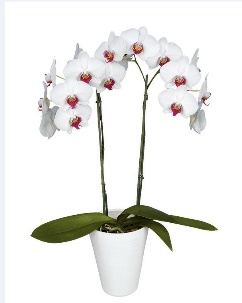 | Similar winter and summer growth rate and development | 67.58 | 0.098 | 1.90 | 66.59 |
| 5 | Purple | 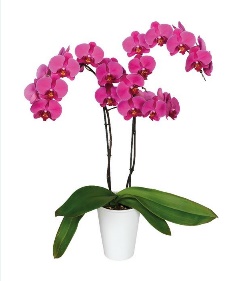 | Similar winter and summer growth rate and development | 68.86 | 0.093 | 1.80 | 65.00 |
| 6 | Yellow | 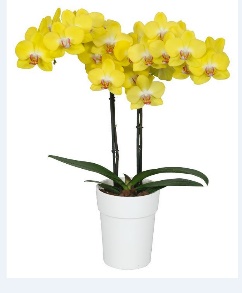 | Slow grower, dark green leaves | 73.55 | 0.086 | 1.88 | 54.47 |
| 7 | White | 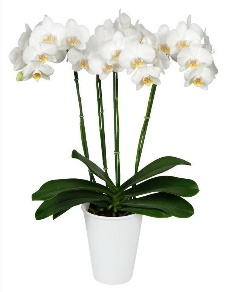 | Fast grower, high number of flower spikes (reduced cooling phase). High leaf initiation rate, but also sensitive to bud abortion | 66.31 | 0.121 | 2.89 | 60.97 |
| 8 | Red | 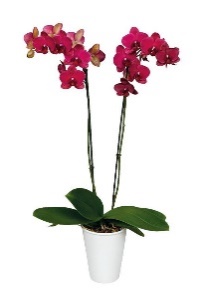 | Sensitive to light, slow growth rate of young plants. Sensitive to leaf damage in winter | 69.49 | 0.099 | 1.94 | 64.81 |
| 9 | Red-copper | 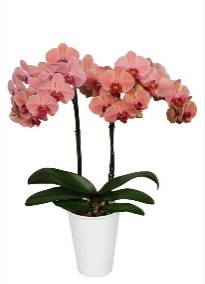 | Sensitive to premature flowering. Leaves have hint of red, anthocyans | 68.24 | 0.101 | 1.80 | 62.50 |
| 10 | Pink | 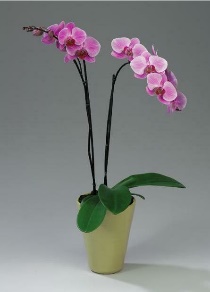 | Few, large leaves. High number of flower spikes. Highly responsive to changes in light quality | 67.38 | 0.106 | 2.09 | 68.42 |
| 11 | Purple | 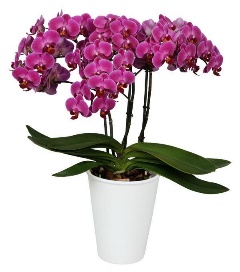 | Multiflora. Flower spikes show high amount of branching. | 68.41 | 0.124 | 2.55 | 44.64 |
| 12 | White | 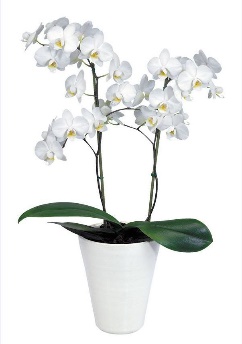 | Multiflora. Small leaves with the tendency to variegate. Relatively large roots | 67.91 | 0.102 | 2.22 | 55.36 |
| 13 | Lilac | 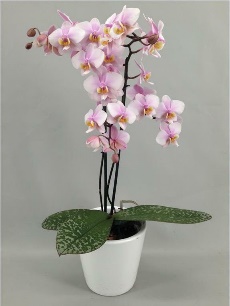 | Multiflora. Variegated leaves, botanical background | * | * | * | * |
| 14 | White | 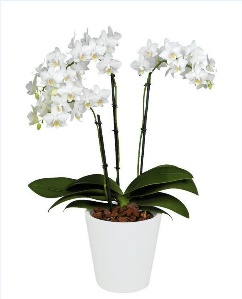 | Multiflora | 58.86 | 0.136 | 2.60 | 50.00 |
| 15 | White with pink stripe | 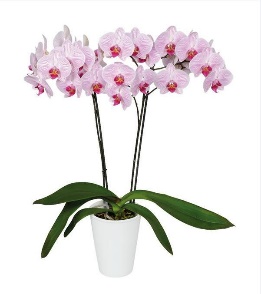 | Sensitive to leaf damage in cooling phase | 67.98 | 0.093 | 1.96 | 64.00 |
| 16 | Pink | 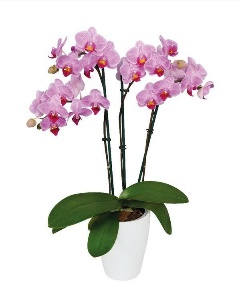 | Multiflora | 69.46 | 0.127 | 2.43 | 50.00 |
| 17 | White | 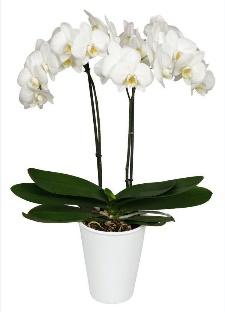 | Plants have average flower size | 65.54 | 0.149 | 2.23 | 50.50 |
| 18 | Pink | 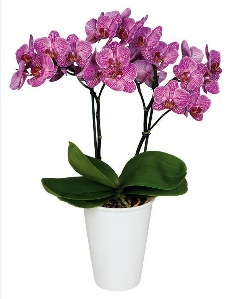 | Very robust and though leaves, with a lighter shade of green. Opposite of genotype 4 and 6, which have leaves that are darker coloured and the tendency to produce antocyanins. | 71.56 | 0.085 | 1.92 | 43.85 |
| 19 | Lilac | 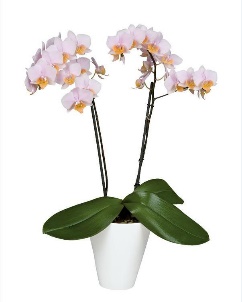 | Often variegated leaves | 68.77 | 0.114 | 1.94 | 52.08 |
| 20 | Yellow | 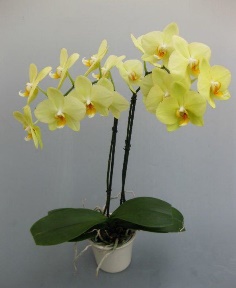 | Low leaf initiation rate, resulting in low number of leaves | 72.38 | 0.085 | 1.97 | 61.25 |
|  |  |  | Average | 68.43 | 0.104 | 2.13 | 58.98 |
