# Supplementary material for: Vegetative traits can predict flowering quality in Phalaenopsis orchids despite large genotypic variation in response to light and temperature
Source: PLoS One. 2021 May 11;16(5):e0251405. doi: 10.1371/journal.pone.0251405 (PMC8112652; doi:10.1371/journal.pone.0251405)
Supplement: S2 File — Genotypic similarity in this study was determined based on the variety tracer method, developed by NAKtuinbouw (Roelofarendsveen, The Netherlands) to identify plant Phalaenopsis varieties. This is done based on the presence or absence of different alleles for 8 SSR markers (H. Teunissen, personal communication). This information was then used to create a similarity matrix based on the Jaccard coefficient. To further display genotypic variation, PCA was done and a dendrogram created that shows the variation of the genotypes used in this study relative to the complete genotypic pool of the breeder, from which plants were acquired. (DOCX) [file pone.0251405.s002.docx]

**S2 File. Genotypic variation**

**2.1 Similarity matrix**

Genotypic similarity in this study was determined based on the variety tracer method, developed by NAKtuinbouw (Roelofarendsveen, The Netherlands) to identify plant Phalaenopsis varieties. This is done based on the presence or absence of different alleles for 8 SSR markers [1][H. Teunissen, personal communication]. Alleles were dominantly scored (based on the presence/absence of polymorphic DNA fragments). In case of doubt during scoring bands were scored as ‘uncertain’, which means that the allele is considered as neither present nor absent. These scores were ignored in the statistical genetic analysis.

In order to investigate the genetic relationship of samples, a data set of absent/present marker scores was generated in a score table. For a simplified representation of these results, techniques such as clustering and ordination analyses are generally employed. The predecessor of these analyses is the construction of a similarity (or distance) matrix. The genetic similarity is the proportion of molecular markers (values varying from 0 to 1 or 100%) that are shared between the two samples being compared.

For construction of the complete matrix all samples are compared to all samples. Several different coefficients have been proposed [2] but for the use of molecular markers similarity coefficients specific for binary variables (presence/absence) are suggested. The similarity matrix was calculated applying the most commonly used  ‘Jaccard’ (*a/n-d*) coefficient [2–4]. BioNumerics’ software (Applied Maths, Sint-Martens-Latem, België ) was used to produce a similarity matrix. The Jaccard coefficient is the international standard for similarity calculations based on binary data.

a, b, c, d, m, n, and u are defined as follows for a two-way frequency table comparing two samples i and j. + present markers; - absent markers.

*m=a+d* (number of matched)

*u=b+c* (number of un-matched)

*n=u+m* (total sample size)

|  | J | | |
| --- | --- | --- | --- |
| I |  | + | - |
|  | + | a | b |
|  | - | c | d |

Jaccard = *a/(n-d)*

| Genotype | | 1 | 2 | 3 | 4 | 5 | 6 | 7 | 8 | 9 | 10 | 11 | 12 | 13 | 14 | 15 | 16 | 17 | 18 | 19 | 20 |
| --- | --- | --- | --- | --- | --- | --- | --- | --- | --- | --- | --- | --- | --- | --- | --- | --- | --- | --- | --- | --- | --- |
| 1 |  | 100 | 100 |  |  |  |  |  |  |  |  |  |  |  |  |  |  |  |  |  |  |
| 2 |  | 100 | 100 |  |  |  |  |  |  |  |  |  |  |  |  |  |  |  |  |  |  |
| 3 |  | 30 | 30 | 100 |  |  |  |  |  |  |  |  |  |  |  |  |  |  |  |  |  |
| 4 |  | 30 | 30 | 27 | 100 |  |  |  |  |  |  |  |  |  |  |  |  |  |  |  |  |
| 5 |  | 33 | 33 | 22 | 13 | 100 |  |  |  |  |  |  |  |  |  |  |  |  |  |  |  |
| 6 |  | 26 | 26 | 19 | 10 | 13 | 100 |  |  |  |  |  |  |  |  |  |  |  |  |  |  |
| 7 |  | 19 | 19 | 17 | 24 | 10 | 11 | 100 |  |  |  |  |  |  |  |  |  |  |  |  |  |
| 8 |  | 32 | 32 | 25 | 11 | 22 | 20 | 8 | 100 |  |  |  |  |  |  |  |  |  |  |  |  |
| 9 |  | 24 | 24 | 29 | 24 | 33 | 30 | 13 | 27 | 100 |  |  |  |  |  |  |  |  |  |  |  |
| 10 |  | 32 | 32 | 21 | 24 | 23 | 13 | 22 | 21 | 16 | 100 |  |  |  |  |  |  |  |  |  |  |
| 11 |  | 20 | 20 | 30 | 9 | 16 | 21 | 10 | 31 | 28 | 23 | 100 |  |  |  |  |  |  |  |  |  |
| 12 |  | 38 | 38 | 20 | 28 | 18 | 24 | 21 | 30 | 26 | 36 | 17 | 100 |  |  |  |  |  |  |  |  |
| 13 |  | 9 | 9 | 11 | 6 | 9 | 0 | 10 | 10 | 6 | 12 | 12 | 10 | 100 |  |  |  |  |  |  |  |
| 14 |  | 15 | 15 | 10 | 12 | 24 | 4 | 18 | 8 | 7 | 11 | 10 | 8 | 11 | 100 |  |  |  |  |  |  |
| 15 |  | 24 | 24 | 15 | 25 | 13 | 13 | 10 | 22 | 19 | 28 | 12 | 22 | 6 | 7 | 100 |  |  |  |  |  |
| 16 |  | 33 | 33 | 31 | 25 | 23 | 26 | 23 | 32 | 29 | 32 | 33 | 33 | 19 | 13 | 14 | 100 |  |  |  |  |
| 17 |  | 38 | 38 | 19 | 27 | 21 | 10 | 30 | 7 | 21 | 21 | 13 | 19 | 6 | 12 | 13 | 16 | 100 |  |  |  |
| 18 |  | 33 | 33 | 30 | 17 | 29 | 32 | 7 | 27 | 38 | 23 | 32 | 27 | 6 | 11 | 20 | 28 | 17 | 100 |  |  |
| 19 |  | 16 | 16 | 21 | 16 | 16 | 9 | 22 | 13 | 18 | 27 | 9 | 21 | 12 | 15 | 19 | 14 | 25 | 19 | 100 |  |
| 20 |  | 52 | 52 | 37 | 23 | 27 | 29 | 17 | 30 | 30 | 22 | 26 | 35 | 9 | 14 | 23 | 27 | 20 | 27 | 18 | 100 |

**2.2 Dendrogram**

In order to further display genotypic variation, a dendrogram was created that shows the variation of the genotypes used in this study relative to the complete genotypic pool of the breeder, from which plants were acquired. To visualize the relationship between the samples a dendrogram was generated using UPGMA parameters (Unweighted Pair-Group Method, Arithmetic average). This kind of algorithms find successive clusters using previously established clusters. Two steps are performed repeatedly: 1. find and merge the two best matches and 2. update the similarity matrix by averaging the scores.

Dendrogram showing 278 examined *phalaenopsis* samples based on the score of 166 alleles using the ‘Jaccard’ similarity coefficient and UPGMA analysis. On the horizontal axis, the similarity is given.


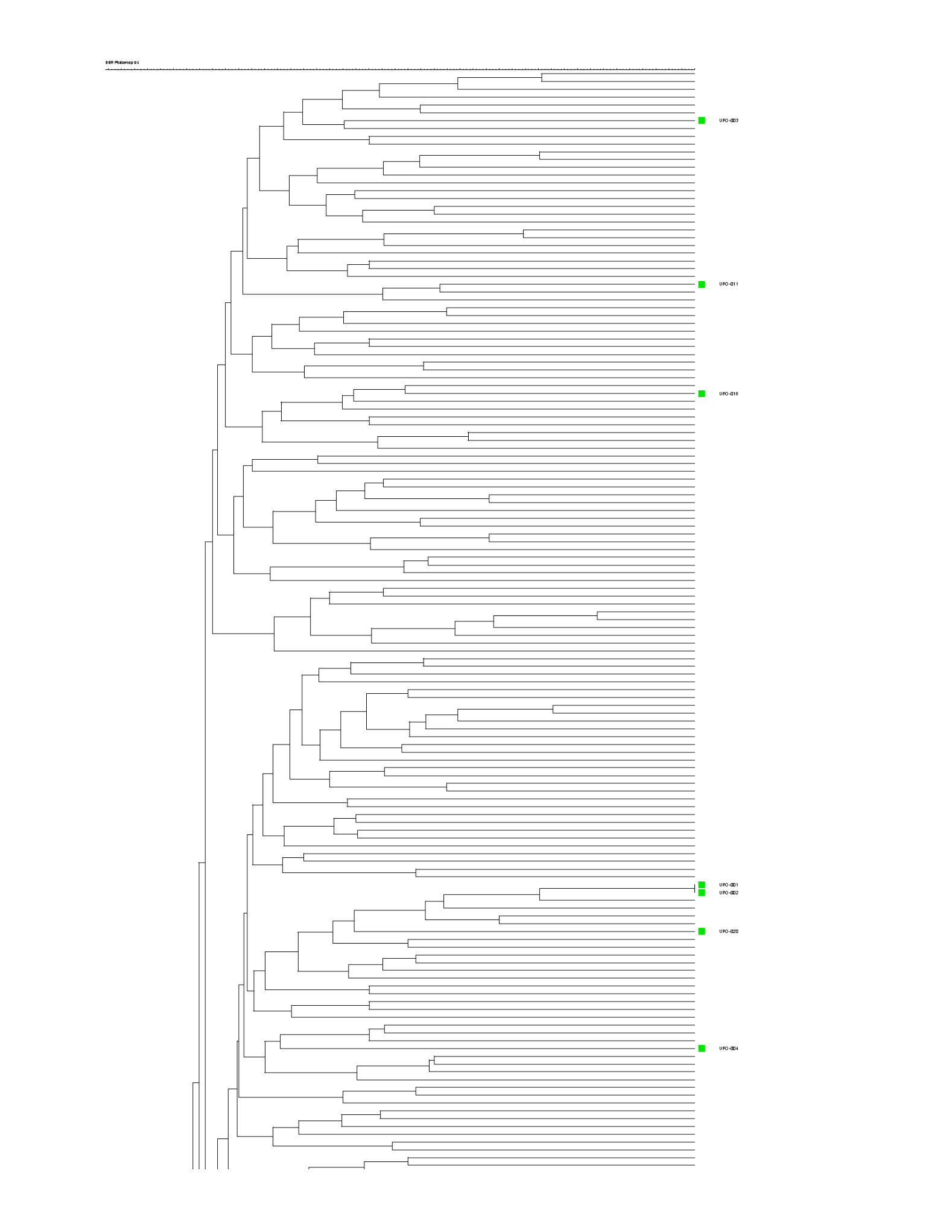


3

11

Genotype

16

1
2

20

4

Continuation of previous page dendrogram


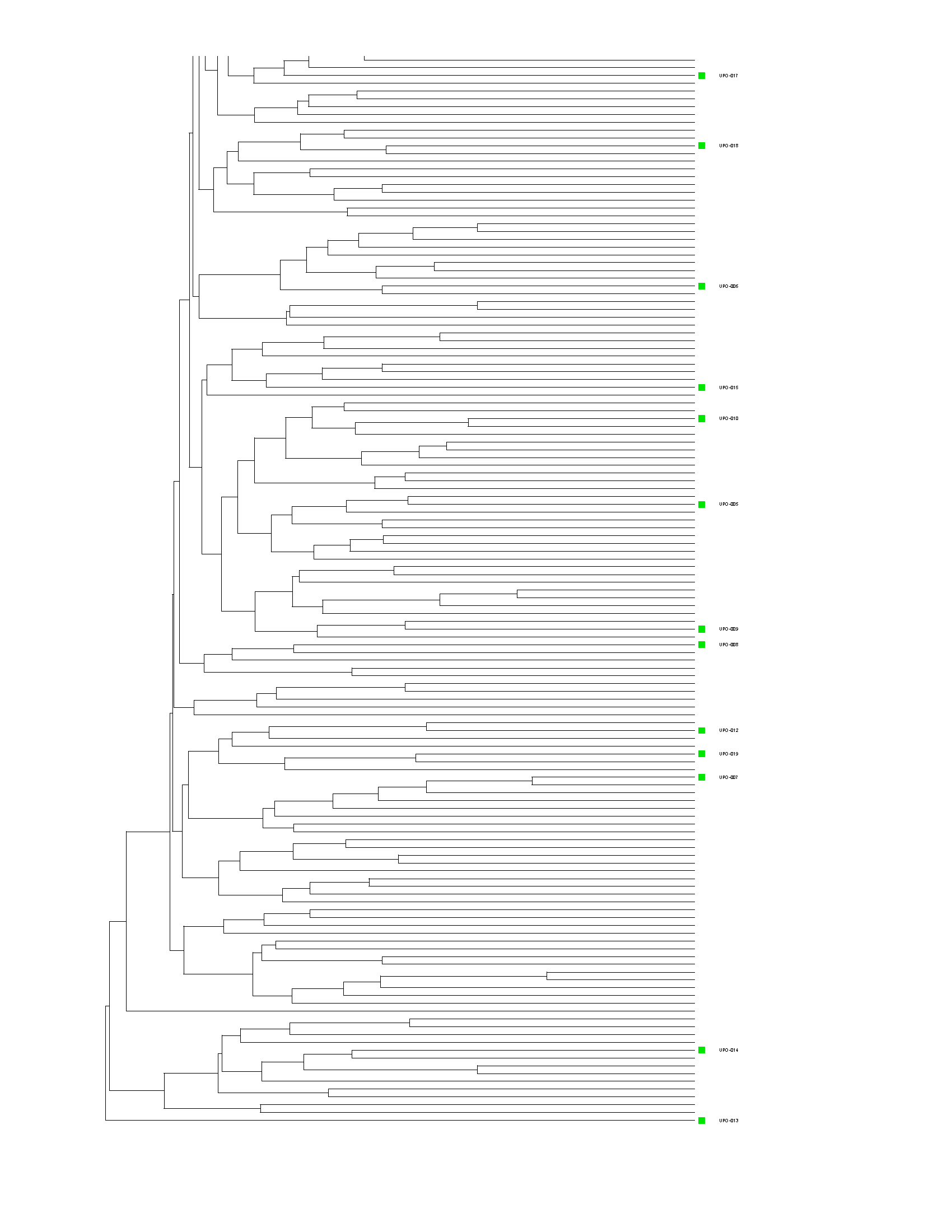


17

18

6

15

10

5

9
8


12
19
7

14

11

13

11

**2.3 PCA**

Principal Component Analysis (PCA) was done on 278 phalaenopsis samples in three dimensions based on 166 alleles. The first dimension explains 6.3% (x- axis), the second 5.5% (y- axis) and the third principal co-ordinate explains 4.7% (z- axis) of the total variation. Coloured group corresponds with the coloured group in the dendrogram.


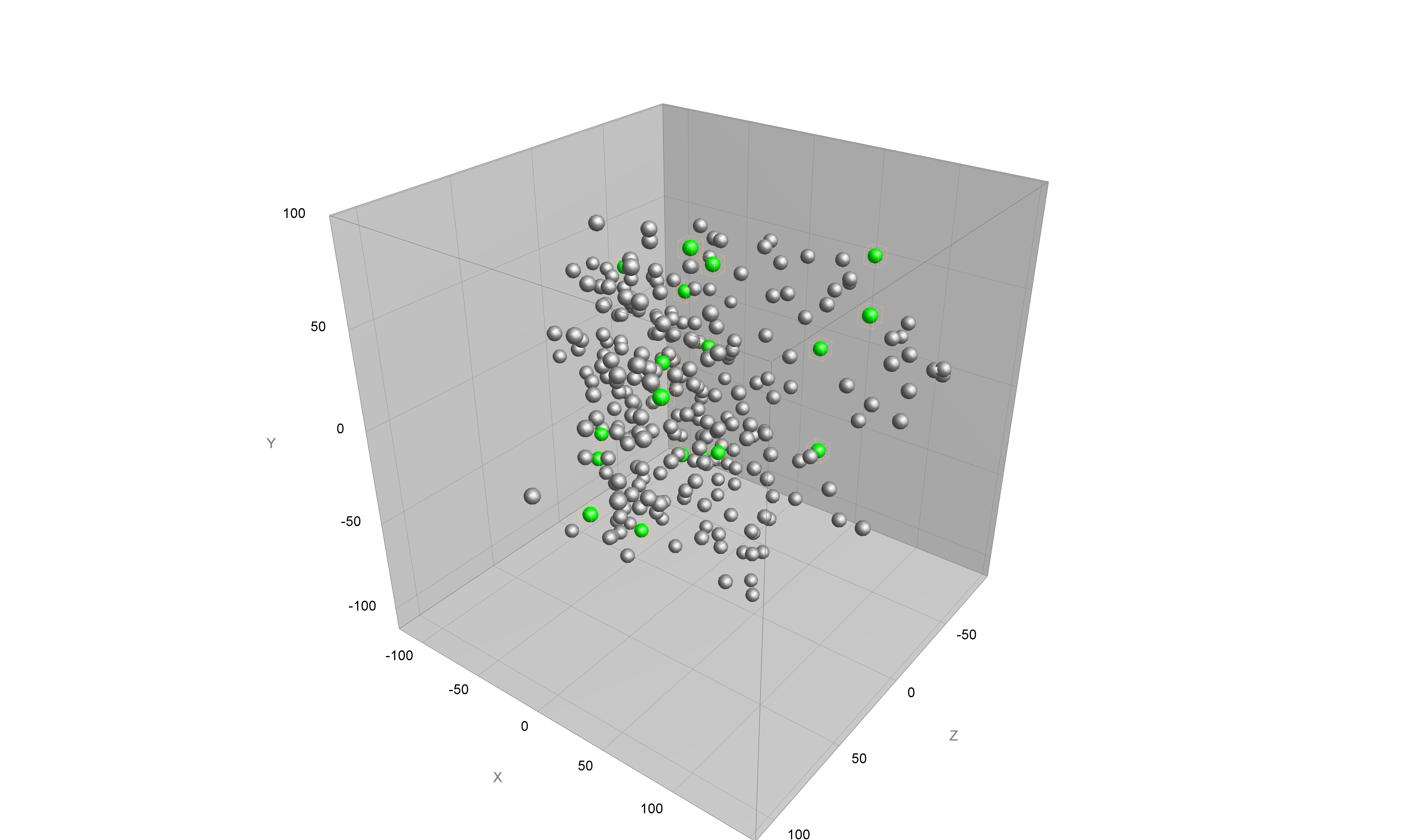


1. Ben-Ari G, Lavi U. Marker-assisted selection in plant breeding. In: Altman A, Hasegawa PM, editors. Plant Biotechnology and Agriculture. First Edit. Elsevier Inc.; 2012. p. 163–84.

2. Sneath PHA, Sokal RR. Numerical taxonomy. The principles and practice of numerical classification. Applied Biostatistics, Inc; 1973.

3. Vierling RA, Nguyen HT. Use of RAPD markers to determine the genetic diversity of diploid, wheat genotypes. Theor Appl Genet. 1992;84(7–8):835–8.

4. Zhang F, Ge Y, Wang W, Shen X, Liu X, Liu J, et al. Genetic diversity and population structure of cultivated bromeliad accessions assessed by SRAP markers. Sci Hortic (Amsterdam). 2012;141:1–6. Available from: http://dx.doi.org/10.1016/j.scienta.2012.04.017
