# Supplementary material for: Vegetative traits can predict flowering quality in Phalaenopsis orchids despite large genotypic variation in response to light and temperature
Source: PLoS One. 2021 May 11;16(5):e0251405. doi: 10.1371/journal.pone.0251405 (PMC8112652; doi:10.1371/journal.pone.0251405)

**S3 File Genotypic variation in flowering *Phalaenopsis* based on plant type**

Breeding in *Phalaenopsis* focusses on creating either smaller plants with a high number of small flowers, or regular sized plants with fewer, but larger flowers and buds; referred to as Multiflora (light grey) and Grandiflora (dark grey), respectively (Fig 1). During the vegetative phase, plants were grown in climate chambers for 15 weeks at either 26°C or 30°C and a PPFD of 60 or 140 µmol m^-2^ s^-1^ for 14 hours per day. Plants from all treatments were simultaneously moved to the greenhouse for cooling and flowering phase until auction-ready; see material and methods – experiment II for details. Average relative changes per genotype per trait to either vegetative reference light intensity (60 µmol m^-2^ s^-1^)(A) temperature (26°C)(B)(n=10).


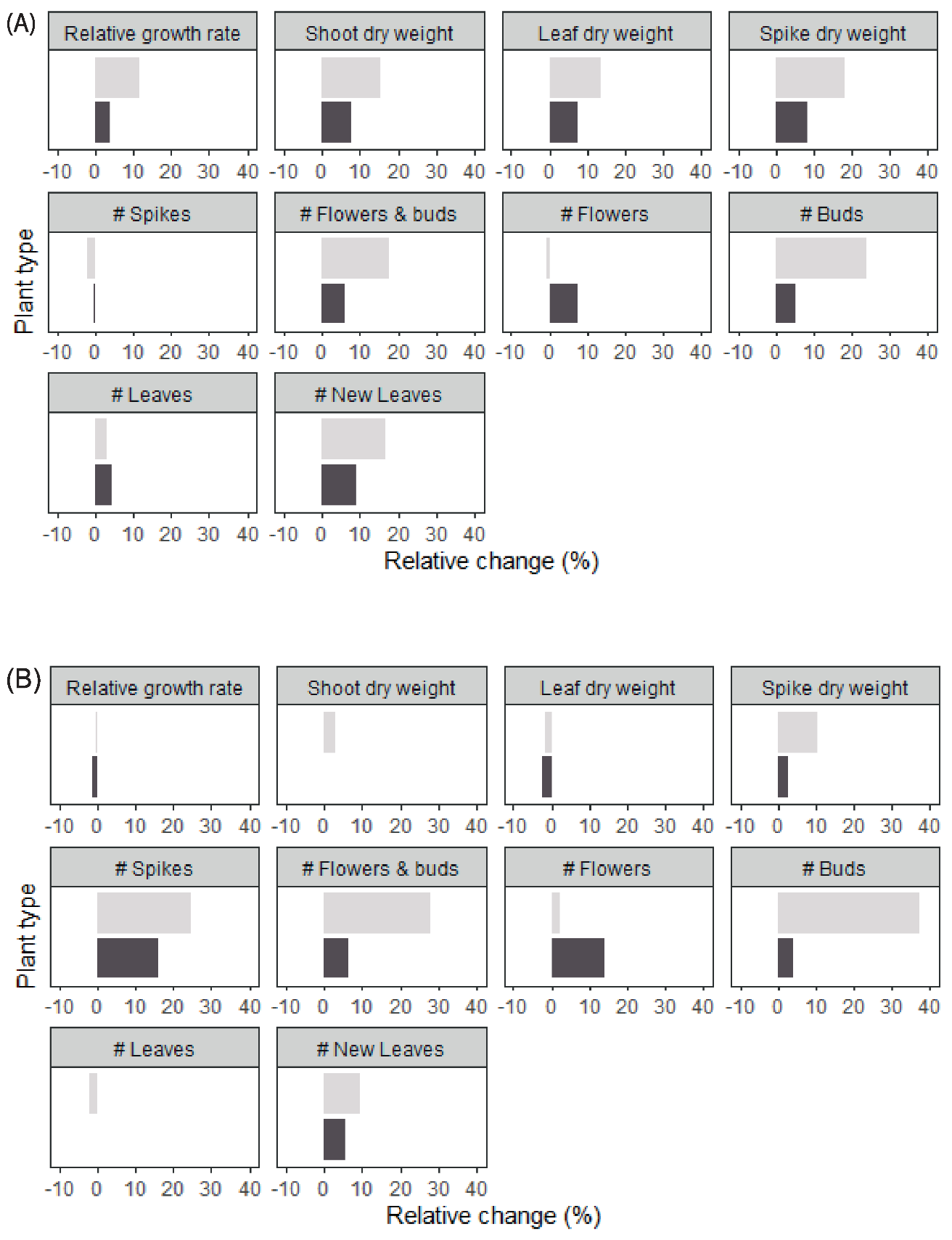

Supplement: S3 File — (DOCX) [file pone.0251405.s003.docx]
