# Supplementary material for: Vegetative traits can predict flowering quality in Phalaenopsis orchids despite large genotypic variation in response to light and temperature
Source: PLoS One. 2021 May 11;16(5):e0251405. doi: 10.1371/journal.pone.0251405 (PMC8112652; doi:10.1371/journal.pone.0251405)
Supplement: S1 Table — (DOCX) [file pone.0251405.s004.docx]

**S1 Table. Composition of nutrient solution**

Plants were watered with a nutrient solution (EC 1.2 mS cm^-1^ and pH of 5.7) composed of the following:

| *Table S3 Composition of nutrient solution* | | |
| --- | --- | --- |
| Ion | | Concentration |
| Macro | N-NO_3_ | 5.6 |
| (mmol l^-1^) | N-NH_4_ | 1.2 |
|  | ‎CH_4_N_2_O | 7.4 |
|  | P | 1.4 |
|  | K | 3.5 |
|  | Ca | 1.3 |
|  | Mg | 0.7 |
|  | SO_4_ | 0.7 |
| Micro | Fe | 36.7 |
| (µmol l^-1^) | Mn | 8.2 |
|  | Zn | 1.9 |
|  | B | 15.1 |
|  | Cu | 1.9 |
|  | Mo | 0.8 |
